# Nested coevolutionary networks shape the ecological relationships of ticks, hosts, and the Lyme disease bacteria of the *Borrelia burgdorferi* (*s.l.*) complex

**DOI:** 10.1186/s13071-016-1803-z

**Published:** 2016-09-23

**Authors:** Agustín Estrada-Peña, Hein Sprong, Alejandro Cabezas-Cruz, José de la Fuente, Ana Ramo, Elena Claudia Coipan

**Affiliations:** 1 Department of Animal Health, Faculty of Veterinary Medicine, University of Zaragoza, Miguel Servet, 177, Zaragoza, Spain; 2 National Institute for Public Health and the Environment (RIVM), Antonie van Leeuwenhoeklaan 9, 3721 MA Bilthoven, The Netherlands; 3 Center for Infection and Immunity of Lille (CIIL), INSERM U1019 – CNRS UMR 8204, Université Lille Nord de France, Institut Pasteur de Lille, Lille, France; 4 SaBio, Instituto de Investigación de Recursos Cinegéticos IREC-CSIC-UCLM-JCCM, Ronda de Toledo s/n, 13005 Ciudad Real, Spain; 5 Department of Veterinary Pathobiology, Center for Veterinary Health Sciences, Oklahoma State University, Stillwater, OK 74078 USA

**Keywords:** *Borrelia burgdorferi* (*s.l.*), Ticks, Vertebrates, Ecological networks, Centrality, Phylogenetic diversity

## Abstract

**Background:**

The bacteria of the *Borrelia burgdorferi* (*s.l.*) (BBG) complex constitute a group of tick-transmitted pathogens that are linked to many vertebrate and tick species. The ecological relationships between the pathogens, the ticks and the vertebrate carriers have not been analysed. The aim of this study was to quantitatively analyse these interactions by creating a network based on a large dataset of associations. Specifically, we examined the relative positions of partners in the network, the phylogenetic diversity of the tick’s hosts and its impact on BBG circulation. The secondary aim was to evaluate the segregation of BBG strains in different vectors and reservoirs.

**Results:**

BBG circulates through a nested recursive network of ticks and vertebrates that delineate closed clusters. Each cluster contains generalist ticks with high values of centrality as well as specialist ticks that originate nested sub-networks and that link secondary vertebrates to the cluster. These results highlighted the importance of host phylogenetic diversity for ticks in the circulation of BBG, as this diversity was correlated with high centrality values for the ticks. The ticks and BBG species in each cluster were not significantly associated with specific branches of the phylogeny of host genera (*R*^2^ = 0.156, *P* = 0.784 for BBG; *R*^2^ = 0.299, *P* = 0.699 for ticks). A few host genera had higher centrality values and thus higher importance for BBG circulation. However, the combined contribution of hosts with low centrality values could maintain active BBG foci. The results suggested that ticks do not share strains of BBG, which were highly segregated among sympatric species of ticks.

**Conclusions:**

We conclude that BBG circulation is supported by a highly redundant network. This network includes ticks with high centrality values and high host phylogenetic diversity as well as ticks with low centrality values. This promotes ecological sub-networks and reflects the high resilience of BBG circulation. The functional redundancy in BBG circulation reduces disturbances due to the removal of vertebrates as it allows ticks to fill other biotic niches.

**Electronic supplementary material:**

The online version of this article (doi:10.1186/s13071-016-1803-z) contains supplementary material, which is available to authorized users.

## Background

The bacteria of the complex *Borrelia burgdorferi* (*sensu lato*) (BBG) are a group of related tick-transmitted pathogens. They are distributed all over the Holarctic biogeographical region [1], with a recent record from South America [2]. A vast range of vertebrates (lizards, birds and mammals) acts as reservoirs for these pathogens. Vertebrates also act as hosts of the ticks that can circulate the pathogens among animals and transmit them to humans [3]. This group of pathogens has been revised considerably in the last decade after the discovery of new strains by phylogenetic, ecological and clinical studies revealed important distinctions among the strains [1, 4, 5]. There is major interest in understanding the ecological processes that lead to infection by a particular species, in the factors that influence the interactions of the reservoirs, their vectors and the prevailing climate [6–8] and in the molecular features that delineate the geographical distribution of the pathogens and their spread [9–11]. There have also been advances in determining the phylogeny of the group using molecular markers [12] and multilocus sequence typing of housekeeping genes [9]. These advances have furthered our knowledge of the complex processes underlying the distribution and the speciation of the pathogens. However, there has not been a major analysis of the ecological relationships of the species of *Borrelia*, the ticks in which they were recorded and the vertebrates that host the bacteria or feed the ticks.

Interactions among species shape biodiversity as communities of organisms that interact and co-evolve across time and space [13–16]. Recent studies have stressed the need to capture the co-evolutionary mechanisms of large sets of interacting species, since a local view or a focus on small groups of species does not encompass their context [17, 18]. The investigation of interacting organisms has produced a corpus, termed the network analysis, that describes the relationships and that stresses the critical need to understand both the persistence and the co-evolution of large assemblages of species in which diversity is the rule [17, 19]. Network theory can describe ecological communities and the distribution of species among the communities and can provide tools to quantify their interactions [20]. Notably, the complexity of a set of interacting species in a network approaches the complexity of the ecological background when there are multiple species in a community [21]. In this context, the interactions between parasites and vertebrates can be considered to be co-evolved structures in networks, a view that supersedes the classic interpretation of diffuse multi-specific interactions [22]. The biological interactions of vertebrates, parasites and transmitted pathogens can thus be depicted as a web in which the nodes represent the organisms and the edges or links represent their interactions [23]. In this approach, the properties of the system emerge from the properties of the organisms, allowing the system to be investigated at both the organismal and the network levels [24]. Accordingly, the extent and the co-occurrence properties of pathogens, ticks and vertebrates can reveal ecological properties that impact the persistence of these complex networks. Generally speaking, networks are characterised by distinct network subgroups (clusters or modules) that are composed of organisms that interact preferentially among themselves rather than with other nodes in the network. In host-parasite networks, closely related host species comprise clusters, in part because they have similar characteristics that determine their compatibility with parasites [25].

The nature of the associations is largely unexplored in tick-transmitted pathogen systems [23, 26, 27]. In this study, we hypothesised that the structure of the network of BBG and its vertebrate hosts and vectors can be investigated using methods derived from network theory. This approach would show the ecological associations and relationships that influence the circulation of the pathogen in active foci. We examined this hypothesis using modularity, which is a network property that is crucial to the ecology of hosts and parasites [23, 25, 28], as a way to investigate the organisation of a large set of records of vertebrates, ticks and BBG pathogens. We further hypothesised that centrality indices can be used to quantify the roles of the partners in this epidemiological network. We demonstrated that the structure of the networks in which BBG circulates is related to the relationships between ticks and hosts and that these relationships can be described based on their centrality indices.

## Methods

### Literature search

Data on the pair-wise systematic associations of ticks in vertebrates, BBG in vertebrates and BBG in ticks were compiled from a literature review of journals that are searchable in the Thomson Reuters, Scopus and PubMed databases. A “record” was a pair-wise BBG-tick-vertebrate combination at a single geographical site. If the same pair-wise combination was observed at the same site several times (for example, in seasonal collections), it was included only once. However, the same combination of partners at different sites was included once for each site in order to estimate how common that association was, and then the number of reports was used for weighting (see below). The literature search extended from January 1990 to December 2014. It was evident in the initial stages of the bibliographical search that the use of a long series of keywords (i.e. a series based on the names of tick species and vertebrate hosts) would miss relevant studies. We therefore decided to perform a deliberately less stringent query that only included the generic names “*Ixodes*” OR “*Borrelia*” and then to critically evaluate the abstracts in order to manually select the papers that dealt only with ecological information. Papers addressing the clinical presentation, treatment or diagnosis of infections in humans were only included in the dataset if adequate information about the association of a species of tick and BBG was reported. We removed records involving livestock because they are considered accidental hosts, and those records would generate spurious information that would distort the relationships of the community of tick-borne pathogens [23]. There are no compilations of records of BBG detected on ticks while feeding because the bacterial DNA cannot be reliably attributed to either the vertebrate or the vector. It is important to stress that the network structure requires bivalent interactions (i.e. the pathogen must be associated with either the tick or the vertebrate). These associations ignore the effects of the vertebrate, which may transmit the pathogen to co-feeding ticks [29, 30].

We also removed records involving species in the recurrent fever group, with some exceptions that were used as controls for the computations (see below), as well as records involving the ticks and vertebrates associated with this group. We updated the scientific names of BBG species to include the most recent and accepted ones [1–8]. Because of the lack of availability of the original publication at the time of the compilation, data on *Borrelia chilensis* and *Ixodes stilesi* [2] were not included in the analysis; we included only the records for *I. pararicinus* [31]. The few data on *B. ruski* were included, although this species has been shown to be *B. afzelii* with a highly variable IGS locus [11]. The final database contained 10,972 pair-wise interactions among species of vertebrates, BBG and ticks. This is available as Additional file 1.

### Building and exploring the network of interactions

To address the ecological aspects of the relationships among BBG-ticks-vertebrates, we developed a network of biotic interactions. The network depicts a community in which each node represents a species and the link between two nodes represents a relationship. In this particular network, nodes represent “carriers” (ticks or vertebrates) that are linked to “cargo” (ticks or pathogens, since vertebrates may carry either). Thus, the network is directed: each link or edge connects one organism to another in one direction. Host-parasite data are sensitive to the sampling effort [32]. Consequently, the computation of further indices is strongly influenced by the sampling and reporting intensity. To ensure that our findings were robust, we increased the weight of the least sampled species and decreased the weight of the most sampled species. Specifically, we regressed the weight of each edge according to the number of citations of the least sampled species (vertebrate, tick, pathogen) in each edge. This regression was highly significant (linear regression: *R*^2^ = 0.56, *F*_(2,2)_ = 34.99, *P* < 0.0001). Next, we additively rescaled the residuals to be greater than zero, since edges cannot have negative weights [32]. The residuals reflect the number of links relative to the sampling effort assuming that the measure of the sampling effort should be based on the less studied species. We replaced the original weights of the edges with the rescaled residuals and then estimated the indices of centrality [32].

Several metrics have been used to investigate the relationships of nodes in networks. The *weighted degree* (WD) is a simple measure of the number of edges that leave or arrive at a given node, and it provides an estimation of the number of nodes connected to every single node. However, the WD does not reflects the importance of the nodes in the context of the network. A more informative measure can be obtained by extending the definition of WD to include the node strength [33], and we use this measure here to indicate the *circulation capacity* (CC) of the pathogen. The CC is a quantitative extension of the species degree, which is the number of interactions per species in a network. The circulation capacity of a species of tick or vertebrate is defined as the sum of dependences (measured as a function of the number and the weight of each edge) of the BBG species that rely on these partners. That is, the CC is a measure of the importance of the vertebrate or the tick relative to the pathogen.

Centrality measures in ecological networks indicate the presence of high-ranking nodes in the network that have significantly higher-than-average connectivity and/or links that stretch far beyond their local network neighbourhoods. Identifying the most central nodes in the system is an important step in network characterization. To quantitatively account for the role of more central nodes, *betweenness centrality* (BNC) was defined as the number of shortest paths between pairs of nodes that pass through a given node. Therefore, central nodes are part of the shortest paths within the network, but peripheral nodes are not. To be in a central node, a carrier had to be infected by many cargo species that infect many other carriers in the network. The vertebrates with the greatest centrality are super-spreaders [34]. It is intuitive to consider the alternative definition of centrality by looking at the *PageRank* (PR), an index of centrality that recursively assigns a universal rank to nodes based on the importance of the other nodes to which it is linked. The *weighted clustering coefficient* (WCC) is a measure of the degree to which nodes in a graph tend to cluster together. The WCC measures the local group cohesiveness and is defined for any node as the fraction of connected neighbours. Thus, the WCC expresses the statistical level of cohesiveness by measuring the global density of interconnected nodes in the network. Network computations were carried out using *igraph* [35] for R [36], and the ForceAtlas2 algorithm was used to display the network [37].

Because of the high complexity of the network and to provide coherence to the analyses, we restricted further computations to the genera of vertebrates. The purpose of reducing from species to genera is to obtain an overall description of the network, since it was impossible to obtain adequate information of the phylogenetic relationships among the species of hosts (see below). We acknowledge this may introduce some bias in the particular role of a host in supporting the circulation of BBG, but do not change the general indexes of the network [23]. The resulting network has 358 nodes with 288 genera of vertebrates, 50 species of ticks, 20 species of BBG [including *B. burgdorferi* (*s.s.*)] and 1,026 links. The nodes of the networks tended to create tightly knit groups that were characterised by a relatively high density of links, which is greater than the average probability of a tie that is randomly established between two nodes [38]. We calculated the modularity of the network using the Louvaine algorithm [39], obtaining also a measure of its nestedness [17]. It has been demonstrated that networks of parasites and hosts tend to be highly nested, and clusters of these networks include both generalist (parasites with many links) and specialist (parasites with few links, shared with the generalists) [23, 26]. In this case we wanted to know if the nested structure already detected for a large set of ticks and pathogens [23] is already retained in the particular case of BBG. Other than records of the BBG group and its associated ticks and vertebrates, we also kept the records of three groups of ticks, vertebrates and pathogens that were not directly related with BBG. One of these groups of records is *Ixodes lividus*, which is a tick parasite on birds of the genera *Riparia* and *Delichon*, a system in which no BBG species have been found. The second group includes *B. persica* and *Ornithodoros tholozani*. This pathogen does not belong to BBG. The third group includes *B. miyamotoi*, which does not belong to BBG but shares a subset of reservoirs with the complex of target pathogens. As a control of the reliability of calculations, we checked whether these two subsystems were separated from the main clusters and routes of circulation of BBG.

### Calculating the phylogenetic relationships of vertebrates

We aimed to evaluate the relationships between the centrality indices of the ticks in the network, the ticks’ ability to transmit species of BBG and the phylogenetic diversity of the exploited vertebrates. To obtain the phylogenetic relationships of the vertebrates, we queried the Open Tree of Life (http://www.opentreeoflife.org/; accessed 19 Sept 2016). We found information for 240 genera of vertebrates, which is summarised in the phylogenetic tree available as Additional file 2. The tree is a representation of the phylogenetic relationships among the genera of vertebrates without information about the times of divergence.

Phylogenetic diversity was calculated using *Faith’s phylogenetic diversity* (PD) index [40] as the total branch length spanned by the tree, including all of the species of vertebrates in a “community”. The term community is used here to mean all of the genera of vertebrates that are included in one of the network clusters and that are exploited by a species of tick or on which BBG has been recorded. We also calculated the *mean pairwise distance* (MPD) and the *mean nearest taxon distance* (MNTD) as described previously [41]. Null models were generated that randomised the tips of the phylogeny to calculate the significance of the phylogenetic association among ticks, BBG and vertebrates [41]. These calculations are intended to link the indices of the network to the vertebrate’s composition of each cluster.

### Multilocus sequence typing and ecological relationships

Multilocus sequence typing (MLST) is a powerful molecular biology technique that is based on using the sequences of 8 chromosomal housekeeping genes to obtain information on population structure and the evolutionary relationships of BBG [9]. We used MLST to evaluate the significance of the associations among strains of BBG and species of ticks or genera of vertebrates. For the MLST analysis, we used the sequences in the *Borrelia* MLST databases (http://pubmlst.org/borrelia; accessed 19 Sept 2016). The data were updated with 613 additional sequences that were obtained from a variety of sources in Europe [42]. The sequences of individual housekeeping genes were concatenated, and those that differed by one or more nucleotides were assigned allele numbers. We assembled a total of 1,832 single associations between BBG, 11 species of ticks and 21 species of vertebrates for a total of 704 unique MLST sequences. We removed all of the strains that were isolated from humans, which constituted more than the half of the available data, since our focus was on wild vertebrates and ticks. All sequences were aligned using the ClustalW MEGA 4 algorithm [43], and phylogenetic trees were constructed using the Kimura two-parameter model in MEGA. We focused on the segregation of MLST sequences and the species of tick or vertebrate in which they have been detected. This analysis should be considered a partial analysis because the available data are not as complete as the data for the relationships of ticks, vertebrates and BBG. All MLST data and data on the ticks or vertebrates in which the pathogens have been reported was translated into a network in which each unique MLST sequence is associated with ticks or vertebrates. We calculated the centrality indices, Faith’s PD, MPD and MNTD as described previously [40, 41]. These are all measures of the phylogenetic diversity of the BBG as calculated for each species of tick or vertebrate, and the aim was to look for a higher-than-expected phylogenetic clustering of strains of BBG with specific vertebrate or tick taxa. Null models were generated by randomizing the tips of the phylogeny to calculate the significance of the phylogenetic association.

## Results

### BBG circulates in a redundant and nested network

The network that supports the circulation of BBG includes 20 species of pathogens of the complex, 18 species of tick vectors and 32 other species of ticks in which the pathogens have not yet been reported but which share vertebrate hosts with those in which the bacteria have been reported. The network also has 82 genera of vertebrate reservoirs of BBG and 206 genera of vertebrates on which the pathogen has not yet been detected but which have been reported as hosts of one or several species of ticks. The network includes 687 species of vertebrates, with 1,026 unique pairs of associations between genera of vertebrates, ticks and BBG. The network is thus highly redundant in terms of vertebrates: several species of ticks feed on the same genus of vertebrate, and the same species of tick may feed on several genera of vertebrates.

**Fig. 1:**
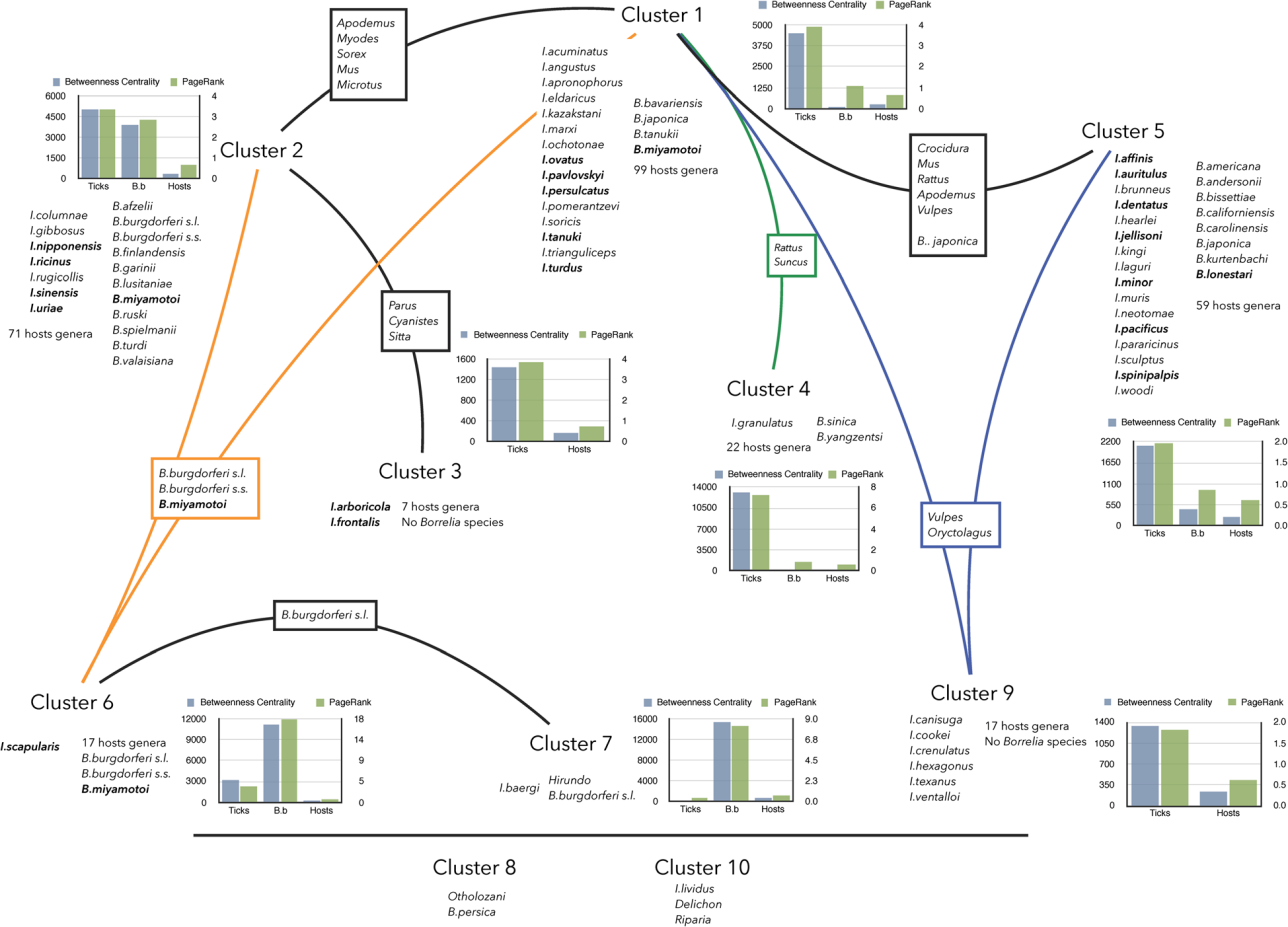
A summary of the epidemiological network of *Borrelia burgdorferi* (BBG), vertebrates and ticks at the resolution of vertebrate genera. The complete network is available in Additional file 2. The clusters detected in the network are numbered randomly. Each cluster shows the species of ticks and the species of *B. burgdorferi* that form the cluster as well as the number of genera of vertebrates that are restricted to each cluster. Bar charts show the betweenness centrality and PageRank values for ticks, BBG and vertebrates. The coloured lines show the articulation points of each cluster, which may include species of pathogens or vertebrates (included as genera). Clusters 8 and 10 were used as controls for the computations since they include a species of *Borrelia* that is not included in the *B. burgdorferi* group (cluster 8) and one species of tick that is monoxenous on two genera of birds and in which no BBG have been reported (cluster 10). The species of ticks that are in bold typeface are those in which species of pathogens of the *B. burgdorferi* group have been reported. The species of *Borrelia* that are in bold typeface are those that are not included in the BBG group but that share vertebrates and ticks with BBG

A schematic view of the network clusters is shown in Fig. 1. Additional files 3 and 4 show the complete network at the level of genera of vertebrates and at the level of species of vertebrates, respectively. The network has 10 clusters. Each cluster is a group of species that are more frequently reported to interact with each other than with other species. The clusters are categorical and are numbered consecutively. The nestedness of the complete network is very high, with a score of 5.2 in a possible range of 0–100, where 0 is the maximum nestedness. The clustering algorithms show the differences in the two groups of species that were included as internal controls, referred to in Fig. 1 as clusters 8 and 10. These are not connected to the other clusters because they contain either non-BBG species of *Borrelia* or ticks that are not connected to the spectrum of vertebrates of the BBG network. The remaining 8 clusters contain a variable number of ticks, vertebrates and pathogens. Clusters 1, 2 and 6 have the highest number of ticks, species of BBG and vertebrates and contain the Palaearctic taxa and *I. scapularis.* The most prominent articulation points of clusters 1 and 2 have at least five genera of vertebrates. Cluster 5 is also notable in the network as it contains all of the New World taxa (except for *I. scapularis*) that circulate on 59 genera of vertebrates and 8 species of BBG, including *B. japonica*. Other clusters are only peripherally connected to the main heart of the network. Cluster 3 contains species of ticks that parasitise birds (*I. frontalis* and *I. arboricola*) without the species of BBG associated with that ecological cluster. These ticks seem to be secondary and perhaps opportunistic transmitters of BBG. Cluster 7 contains the specialist tick *I. baergi*, in which BBG has been detected; cluster 9 contains species of carnivores and other mammals, the articulation points of which are vertebrates that are shared with ticks of other clusters and with which no BBG species are ecologically related. Cluster 4 is of special interest because it is strongly associated with both *B. sinica* and *B. yangtzensis*, both of which were reported in the tick *I. granulatus* and in several vertebrates. Clustering algorithms indicate that this cluster only contains species that have been collected in the Palaearctic region. Additional files 5, 6 and 7 include all the numeric data related to ticks, BBG and vertebrates.

### BBG circulates through ticks with high values of centrality and phylogenetically diverse vertebrates

**Fig. 2:**
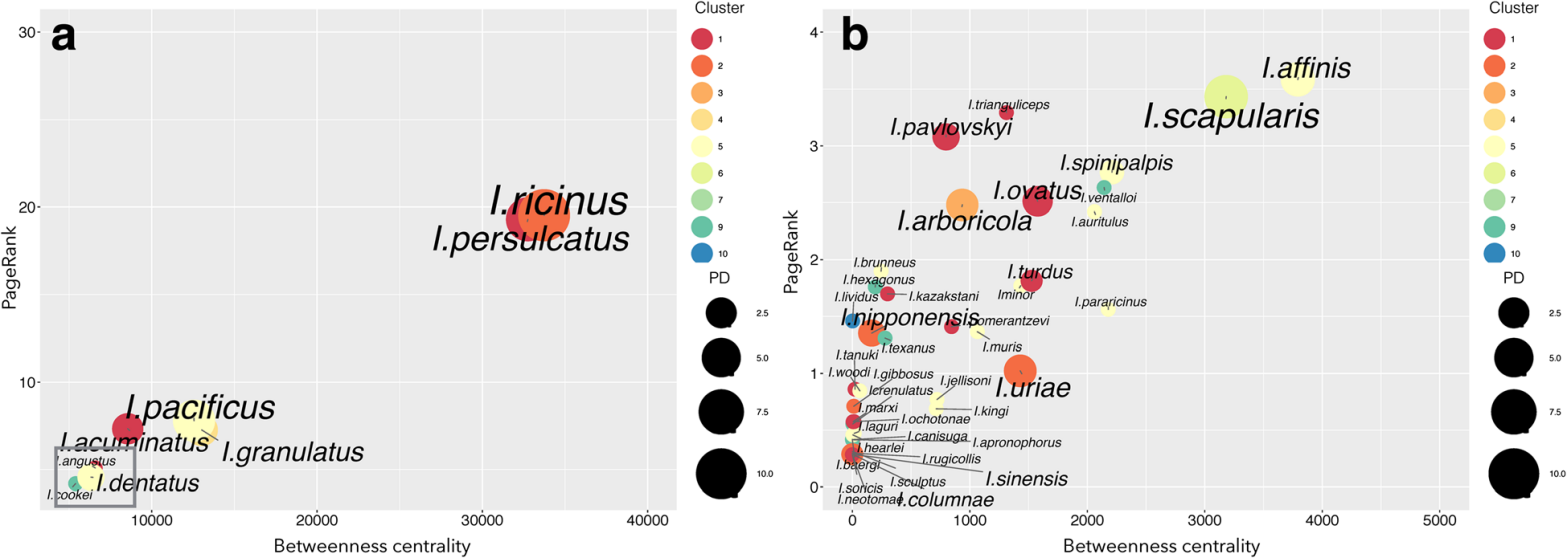
The distribution of the betweenness centrality and PageRank values of the tick species in the epidemiological *Borrelia burgdorferi* (BBG) network. This plot shows the number of clusters in which the ticks are allocated (indicated by the *colours*) and the phylogenetic diversity of the vertebrates of each tick species as measured using Faith’s phylogenetic diversity index (PD; indicated by the *circle size*). **b** shows an expanded view of the species in the square at the bottom left in **a**

**Fig. 3:**
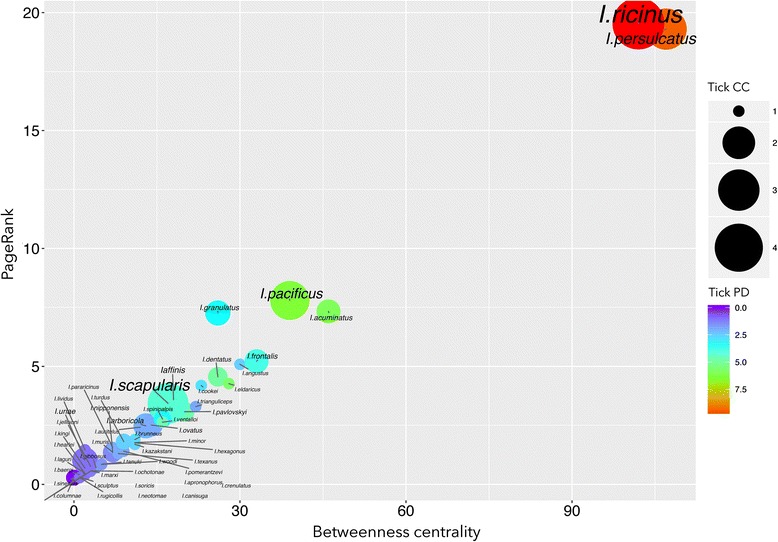
The distribution of the betweenness centrality and PageRank values of the tick species involved in the *Borrelia burgdorferi* (BBG) epidemiological network. This plot shows the circulation capacity of each tick species for BBG (TicksCC, indicated by the *circle* size) and the phylogenetic diversity of the vertebrates of each species of tick as measured using Faith’s phylogenetic diversity index (TicksPD, circle colour)

We wanted to determine whether the ticks in the different clusters had different properties in the context of the network. Figure 2 shows that the ticks in the different clusters were distributed randomly in terms of the range of BNC and PR values and that the Faith’s PD of the hosts of the ticks was correlated with both the BNC and the PR values. Specifically, the ticks with the highest Faith’s PD values tended to occupy the top positions in the network in terms of BNC and PR values (PD-BNC: *R*^2^ = 0.713, *F*_(2,2)_ = 114.57, *P* < 0.0001; PD-PR: *R*^2^ = 0.818, *F*_(2,2)_ = 207.95, *P* < 0.0001). *Ixodes ricinus* and *I. persulcatus* had the highest centrality (>90) and Faith’s PD (18.13 and 5.81, respectively) values, with other prominent species in the transmission cycles of BBG (like *I. scapularis* and *I. pacificus*) showing relatively high Faith’s PD (3.08 and 1.51, respectively) values but low BNC (17 and 35, respectively) and PR (3 and 8, respectively). Only two species of ticks with lower BNC (< 60) and PR (< 1) values have been reported as being infected with BBG (*I. columnae* and *I. sinensis*). All the other reported vectors of BBG had high BNC and PR values. We calculated the CC of BBG by ticks and plotted it against their Faith’s PD and centrality indices. The result was an almost linear relationship (*R*^2^ = 0.799, *F*_(1,2)_ = 27.65, *P* < 0.0001) (Fig. 3). However, this correlation was reduced if Faith’s PD was replaced by the crude number of genera of hosts (*R*^2^ = 0.211, *F*_(1,2)_ = 1.97, *P* = 0.349).

Statistical analysis of the Faith’s PD of vertebrates showed that the clusters were not linked to specific branches of the phylogenetic tree of vertebrates. Rather, each cluster included groups of vertebrates with high Faith’s PD. There were no statistically significant differences in the associations of the ticks or BBG in each cluster with specific branches of the phylogeny of the genera of vertebrates (*R*^2^ = 0.156, *P* = 0.784 for BBG; *R*^2^ = 0.299, *P* = 0.699 for ticks; Additional file 8). Not all species of ticks could be analysed either because reliable phylogenetic data for the vertebrates was lacking or because the ticks were restricted to only one genus of vertebrates. The following tick species were tightly linked to specific branches of the phylogeny of vertebrates: *I. angustus*, *I. arboricola*, *I. cookei*, *I. frontalis* and *I. trianguliceps*. Notably, all of the species of ticks that were restricted to a set of a few vertebrates had low importance in terms of the circulation of BBG, but all of them occurred in clusters in which generalist ticks were predominant. The same calculations were performed using the BBG species and the phylogeny of the vertebrates on which they were reported. The results showed higher phylogenetic diversity of vertebrates for BBG than for ticks, and only *B. carolinensis* and *B. yangtzensis* were linked to a specific branch group of vertebrate genera. Additional file 8 shows the relationships of the organisms in clusters 1, 2, 5 and 6 with the branches of the vertebrate phylogenetic tree.

**Fig. 4:**
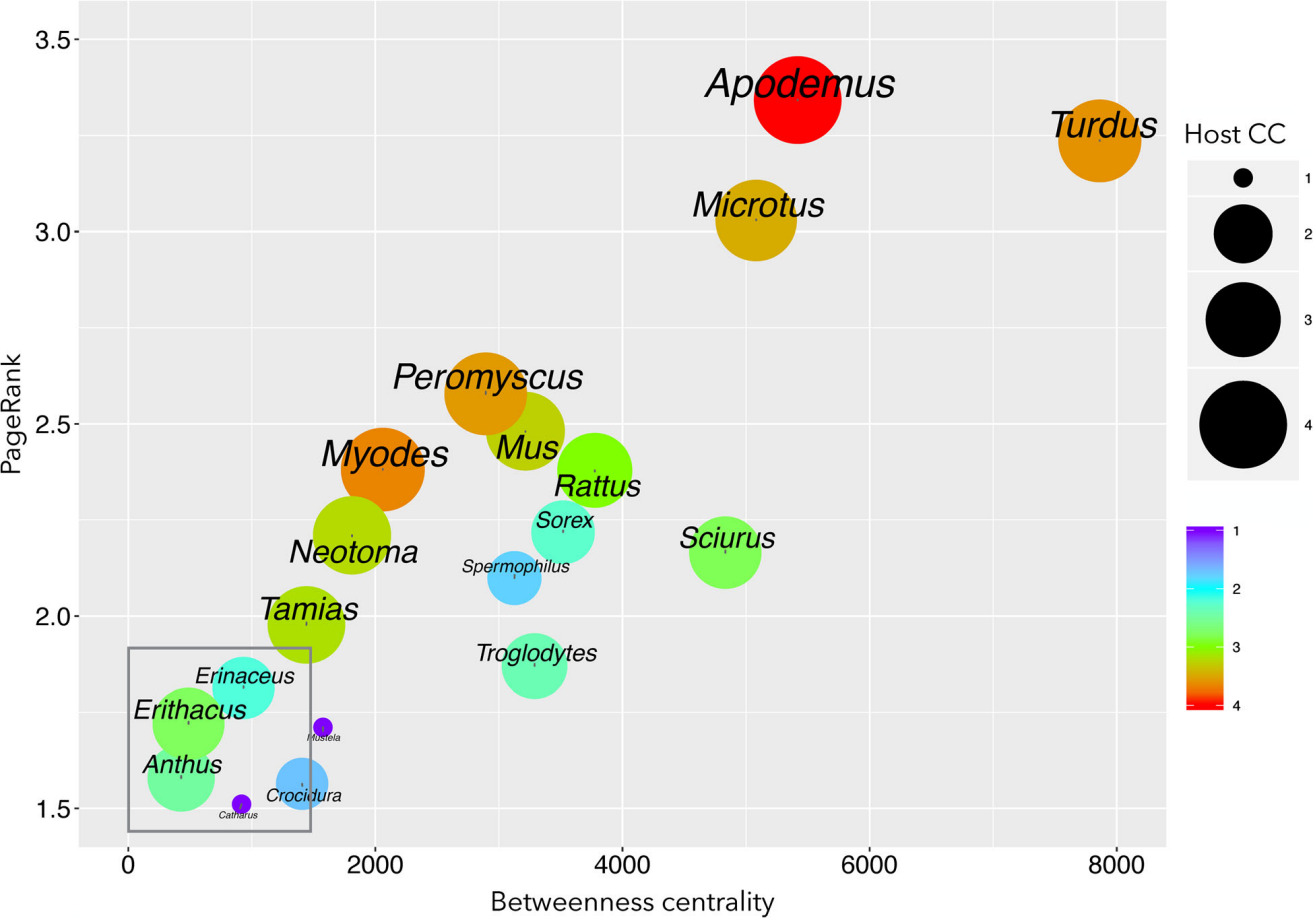
The distribution of the betweenness centrality and PageRank values of the genera of hosts in the *Borrelia burgdorferi* (BBG) epidemiological network. This plot shows the circulation capacity of each host genus for BBG (indicated by the size and colour of the *circle*). An expanded view of the rectangle that contains low values of centrality is shown in Fig. 5

**Fig. 5:**
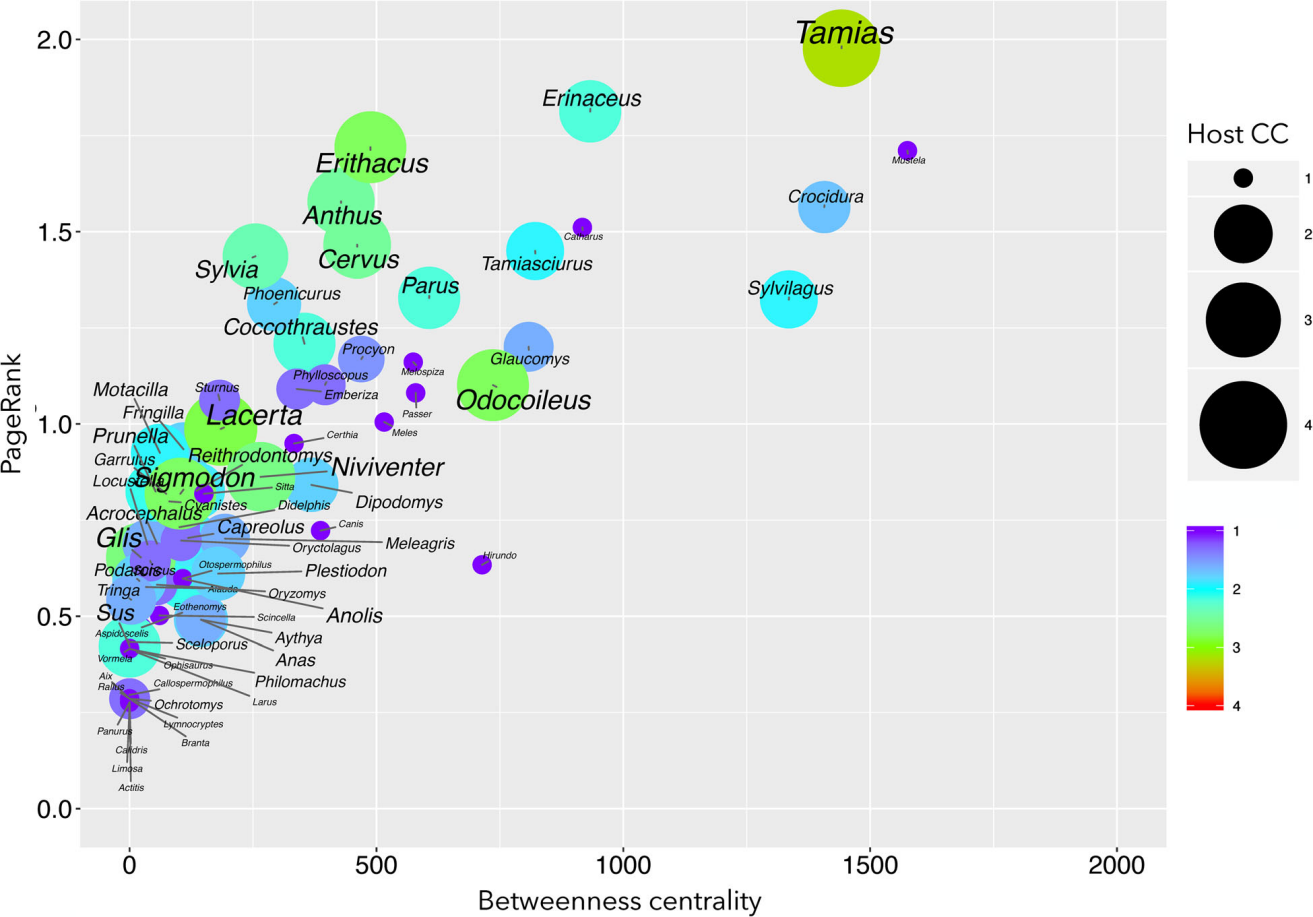
The distribution of the betweenness centrality and PageRank values of the genera of hosts in the *Borrelia burgdorferi* (BBG) epidemiological network. This plot shows the circulation capacity of each host genus for BBG (indicated by the *circle* size and colour). This is an expanded view of the rectangle in Fig. 4

A few genera of rodents and medium-sized birds had some of the highest centrality values of the network. They also had the greatest CC values for BBG. The genera of hosts with the highest centrality index values were *Turdus*, *Apodemus* and *Microtus* (Fig. 4). However, the relationship between the CC and the position of the vertebrate in the range of centrality values was not completely linear (*R*^2^ from multiple regression = 0.633, *F*_(1,2)_ = 11.98, *P* < 0.0001) since some genera of hosts, like *Crocidura* and *Spermophilus*, also had relatively high PR and BNC values and made low contributions to the circulation of BBG. A few hosts with PR and BNC values in the low range might also make important contributions to the circulation of BBG (Fig. 5), such as small, ground-feeding birds (*Erithacus*, *Anthus* and *Sylvia*), a lizard (*Lacerta*) and two large ungulates (*Odocoileus* and *Cervus*). Data from regressions performed using centrality indices and CC suggested that the PR is the variable with the most influence. The ecological implication is that vertebrates with the highest values of centrality for ticks are also those that support the highest circulation of BBG.

### Ticks do not share strains of BBG

**Fig. 6:**
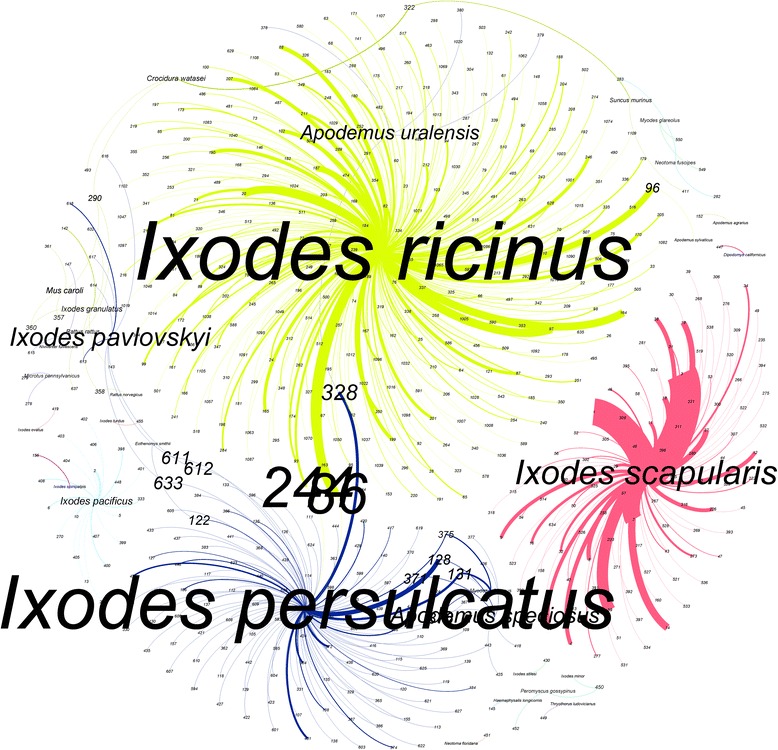
The *Borrelia burgdorferi* (BBG) network as characterised by multilocus sequence typing (MLST) with the associated ticks and vertebrates. The ForceAtlas2 algorithm was used to determine the layout of the clusters. Only BBG strains recorded from questing ticks or vertebrates were included. Each randomly chosen colour represents a cluster, and the label size is proportional to its importance in the context of the network (evaluated as its centrality). The numbers at the end of each link indicate the number of the strain according to the MLST scheme. The width of each link is proportional to the number of times that a given strain has been reported in a given tick or vertebrate

We further evaluated the links between strains of BBG (as defined by MLST analysis) and the taxa of ticks and vertebrates. This test could not be performed for several species of carriers because only one strain was available; therefore, phylogenetic diversity could not be calculated. The results must thus be considered a proof of concept that is still preliminary and incomplete due to the bias in recording and typing strains from the most commonly surveyed ticks and vertebrates. Table 1 shows the phylogenetic diversity of the strains of BBG in the different carriers. Faith’s PD was very low for *I. granulatus* (0.07483), *I. pacificus* (1.517) and *I. pavlosvkyi* (1.991), it was medium for *I. scapularis* (3.0) and very high for *I. ricinus* (18.13). Negative MPD values and values of MPD-P near 0.01 indicate strong phylogenetic clustering of the MLST strains in the carrier species. Although the results should be interpreted cautiously due to the small sample size, we found a strong phylogenetic link between the BBG strains and the tested species of *Ixodes* (0.01 for the species with more than one strain reported) but not *I. ricinus*. Figure 6 shows the network of MLST strains that were linked to unique species of ticks or vertebrates without overlapping BBG strains in the ticks. Additional files 9, 10, 11, 12 and 13 report the MLST-derived phylogenetic tree of the BBG strains and show the links of the ticks to specific portions of the BBG tree. Additional file 14 complements Fig. 4 by labelling the BBG species that were linked to the nodes of ticks or vertebrates. *Ixodes ricinus* and *I. persulcatus* (Palaearctic species) shared only two strains, MLST numbers 86 and 244; however, *I. persulcatus* and *I. pavlovskyi* shared several strains, and these strains were similar to those found in *I. turdus* and *I. granulatus*, which are partially sympatric species. *Ixodes pacificus* (western Nearctic) strains are more similar to strains found in eastern Palaearctic ticks. Fewer strains of BBG have been detected in *I. scapularis* (eastern Nearctic), and they are highly repeated*.* The strains of BBG in *I. scapularis* and *I. pacificus* (Nearctic) were very different in the context of the network.

**Table 1:** The phylogenetic diversity of *Borrelia burgdorferi* (*s.l.*) strains recorded in vertebrates or ticks based on their characterization by multilocus sequence typing (MLST). Negative MPD values and values of MPD-P near 0.01 indicate strong phylogenetic clustering of the MLST strains in the tick or vertebrate; positive MPD values and higher MPD-P values indicate weak phylogenetic clustering of the MLST strains in the tick or vertebrate

Species	PD	*n*	MPD	MPD-P
*Apodemus agrarius*	na	1	na	na
*Apodemus speciosus*	2.86735	8	-2.10119	0.05
*Apodemus sylvaticus*	na	1	na	na
*Apodemus uralensis*	0.13776	4	-3.64226	0.01
*Crocitara watasei*	0.02041	3	-3.08071	0.01
*Dipodomys californicus*	na	1	na	na
*Eothenomys smithii*	na	1	na	na
*Microtus pennsylvanicus*	0.0102	2	-2.40207	0.01
*Mus caroli*	0.06633	4	-4.52009	0.01
*Myodes glareolus*	1.70408	2	0.57406	0.72
*Myodes rufocanus*	0.11224	3	-4.04499	0.01
*Neotoma floridana*	na	1	na	na
*Neotoma fuscipes*	0.0068	2	-2.0568	0.01
*Niviventer fulvescens*	na	1	na	na
*Peromyscus gossypinus*	0.0068	2	-2.19929	0.025
*Rattus norvegicus*	na	1	na	na
*Rattus rattus*	0.04762	2	-2.06397	0.02
*Suncus murinus*	0.01361	2	-2.44465	0.02
*Thryothorus ludovicianus*	na	1	na	na
*Haemaphysalis longicornis*	na	1	na	na
*Ixodes granulatus*	0.07483	6	-5.10505	0.01
*Ixodes minor*	na	1	na	na
*Ixodes ovatus*	na	1	na	na
*Ixodes pacificus*	1.51701	20	-9.68056	0.01
*Ixodes pavlovskyi*	1.9915	9	-5.07941	0.01
*Ixodes persulcatus*	5.81122	116	-6.09055	0.01
*Ixodes ricinus*	18.13265	307	4.61072	1
*Ixodes scapularis*	3.0085	104	-25.75207	0.01
*Ixodes spinipalpis*	na	1	na	na
*Ixodes stilesi*	na	1	na	na
*Ixodes turdus*	na	1	na	na

*Abbreviations*: *MPD* mean pairwise distance, *MPD-P* significance of MPD index, *na* not available, *PD* Faith’s phylogenetic diversity, *n* number of haplotypes recorded

## Discussion

Here we present the largest BBG epidemiological network that has been analysed to date. This network showed that the pathogens circulate in closed clusters in a highly nested manner and that they are supported by a few species of ticks that exploit a range of phylogenetically diverse hosts. These ticks are restricted to closed clusters that closely resemble geographical components. We found that the vertebrates that support BBG circulation are those that are most commonly used as hosts by generalist species of ticks. Specialist ticks interact with a few species of vertebrates to generate sub-networks that link other vertebrates that would otherwise remain unconnected to the main web structure.

The constructed network of pathogens*,* their vertebrate hosts and their vectors formed a structure of redundant interconnections. Some species of BBG linked a few otherwise isolated clusters, suggesting invasion events and further spread; these have been described previously [10]. However, undetermined BBG genospecies were included in the network in order to use data obtained before modern molecular techniques became available, and this could result in bias in terms of the structure of the clusters. A combination of centrality indices was used to quantify the relative importance of each tick in the network, with centrality being positively correlated with the phylogenetic diversity of the species of ticks but not with its host richness. Vertebrates that had the highest centrality with ticks also contributed more to the circulation of the pathogens. Circulation of the pathogens is thus supported by a wide range of vertebrates and by the generalist ticks that they share. The framework revealed the co-occurrences and interactions of specialised and generalised species that are key to maintaining functional diversity [44], promoting community stability [45] and ensuring network persistence [17].

The existence and underlying causes of clusters in ecological networks are still a matter of debate. In parasite networks, the hosts of the most specialised species of tick tend to be a subset of the hosts of the ticks that are slightly more generalised; in turn, these hosts are a subset of the hosts of ticks that are even more generalised, and so on [46]. We tested the efficiency of the cluster-detecting algorithm by retaining the records of (i) species of ticks that shared vertebrate hosts with other ticks in which BBG has been detected; (ii) ticks and vertebrates in which the pathogen has never been reported; and (iii) species of *Borrelia* that are not BBG. In every case, the algorithm assigned the partners to the correct clusters. Specifically, the algorithm placed *B. miyamotoi* (which is not a member of BBG) in a subset of vertebrates that are shared by BBG species, as reported previously [47]; it separated *B. persica* from the circulation of the main network; and it found that *I. lividus* was not involved in BBG circulation. These findings supported other results derived from the network.

The findings from the BBG ecological network agreed with those of other studies of the phylogenetic structure of the populations of other species in BBG. For example, it has been reported that *B. bavariensis* comprises two populations, namely a genetically diverse Asian population and a genetically homogeneous European population. The observed differences in genetic heterogeneity cannot be attributed to different geographic scales of sample origin [48]. For example, other BBG species with a low prevalence or a focal distribution in Europe, such as *B. spielmanii* or *B. lusitaniae*, show genetic heterogeneity according to MLST analysis, while *B. bavariensis* does not [48]. The very low diversity and short branches of the phylogeny of the European population suggest a recent founding event that is probably linked to the shift of a population of *B. bavariensis* to the European vector, *I. ricinus* [48]. Our network results support the idea that *B. bavariensis* is not native to Europe, since it circulates within a cluster of 99 genera of vertebrates and 15 species of ticks that are distributed in the eastern Palaearctic. *Ixodes ricinus,* a Western Palaearctic species*,* is not a “native” tick in that cluster.

This study has some limitations. The network approach ignores the abilities of the vertebrates to acts as reservoirs, a feature that has been evaluated elsewhere using both regional [46, 49, 50] and larger sets of empirical data [11, 51–53]. This approach also ignores transmission inhibition in vertebrates that are infected by several strains [54]. Each vertebrate has different reservoir capacities and thus infects ticks at different rates and contributes in varying ways to the circulation of the pathogen. Since our approach was based on the genera of vertebrates, and since there is a scarcity of experimental data about the reservoir capacity of vertebrates, we could not address these issues in the current study. Another limitation of the study is the phylogenetic construction of the genera of vertebrates. Because of the large number of vertebrate species, the inclusion of taxa from both the Palaearctic and Nearctic regions and the unavailability of data for some species, a complete phylogenetic approach was to use a mega-phylogeny [55, 56]. We eliminated a potential source of errors that commonly arise from literature searches by weighting the number of records [23, 32].

The ecological interpretation of our findings is that groups of allopatric species of ticks delineate clusters of interacting organisms. These clusters are not phylogenetically linked to the genera of vertebrates, indicating that they are not derived purely from the relationships of ticks with specific branches of vertebrate evolution. The results revealed direct relationships between the CC of the pathogen, the phylogenetic diversity of the vertebrates that feed ticks (but not the raw richness of the host taxa) and the ticks’ centrality values. The results also showed the relative importance of some vertebrates for BBG circulation. Notably, the prevalence of BBG in ticks is due to a complex relationship based on the tick densities on available hosts, on the capacity of the hosts to feed the ticks and on the capacity of the hosts to transmit BBG to the feeding ticks [57]. Thus, the tick burdens of vertebrates are positively correlated with the infection prevalence of BBG, suggesting that the majority of ticks are fed by only a few species of hosts. Our results are in line with previous reports linking the life-cycle traits of the hosts with their tick burden and to the infection prevalence of BBG in hosts [52, 58]. Considering our simplification, which included the use of the genera of hosts rather than the species of hosts, the results from the network still overlapped and agreed with previous quantitative reports on the topic. In particular, they highlighted the importance of medium-sized birds, some rodents and Insectivora in supporting active BBG foci. It is important to stress that the same basic ecological structure is found using different methods, including quantitative reviews of the BBG-vertebrate system [57], the life-cycle traits of the hosts [52] and our network, which was constructed using crude association data on ecological relationships. Vertebrates that were identified as being the most important for the circulation of ticks were those with the highest importance for pathogen circulation, an intuitively obvious finding that now has a mathematically tractable framework. It must to be stressed that the circulation of BBG is supported by either the reservoir ability of the hosts and/or the feeding of large number of ticks. This may be the case of some large ungulates, which are hosts of large numbers of ticks but are recognised as inefficient reservoirs of the pathogen.

We speculate, however, that the importance of underrepresented hosts (e.g. those with low centrality) has been neglected. Alone, the individual contributions of hosts in the low range of centrality would probably not be able to support active BBG foci in the absence of the most central vertebrates, but their collective contributions, even with low values of centrality, would support the successful circulation of BBG in biodiverse foci. The network could be revised to integrate previous quantitative approaches into the ecological background in order to further study the influence of vertebrates and ticks in the complex relationships of BBG. Further research on the topic is needed, including the individual contributions of species of hosts (i.e. according to body size and number of feeding ticks) although this seems to be a very local feature, that may change even from year to year according to the relative faunal composition of available hosts and the tick-host relationships observed at a given site.

Our results suggest that the BBG network depends on the persistence of the selective forces required for the coevolution of these assemblages. We postulate that two forces act in combination to lead to the observed architecture of the BBG network: one, functional redundancy (i.e. the many vertebrates and ticks involved in pathogen circulation), with varying degrees of relative importance; and two, the co-evolutionary overlap of the environmental niches of ticks and vertebrates. We postulate that interactions between ticks and vertebrates were initially derived from their complementary ecological requirements, thereby increasing functional redundancy and allowing the pathogens to circulate. If the environmental dimensions of the interacting species expanded, or if new species converged towards such niches due to interactions, then other species became part of the evolving network, leading to the observed sub-networks. We hypothesise that sub-networks are the result of secondary niches between ticks and hosts that led to subdivisions of the central core of the network.

We updated the publicly available MLST dataset and produced 704 unique sequences with the aim of checking the links to ticks and/or vertebrates. These results are preliminary due to bias stemming from the reporting of the pathogens in only a few species of ticks and the scarcity of data from vertebrates. However, it is important to notice that (i) *I. ricinus* (western Palaearctic) and *I. scapularis* (eastern Nearctic) do not share any BBG strains; (ii) *I. persulcatus*, *I. pavlovskyi* and *I. granulatus*, species that partially overlap in their Palaearctic distribution range, share several BBG strains; (iii) *I. pacificus* (western Nearctic) share more strains of BBG with ticks of the eastern Palaearctic; and (iv) the strains of BBG in *I. scapularis* are highly repeated. Phylogenetic diversity calculations corroborate the higher diversity of the BBG strains associated with the most surveyed Palaearctic ticks, *I. ricinus* and *I. persulcatus*. The results show many of the same strains in *I. scapularis*, which are slightly related to the *I. ricinus* and *I. persulcatus* strains, and this could be interpreted as the result of a recent BBG invasive event. It is unknown whether this is the result of the geographical segregation of BBG or whether it is an adaptation of BBG strains to the carriers, since the strains were more similar in ticks that are partially sympatric. It has been suggested that BBG adapts to the hosts they find most frequently, resulting in the observed MLST patterns [57]. However, we hypothesise that the ticks could be an important part of the evolutionary pressure exerted on the housekeeping genes of the pathogens. This would be in agreement with previous reports of the tick-borne pathogens *Anaplasma marginale* [59] and *A. phagocytophilum* [26, 60, 61].

## Conclusions

The network constructed in this study, which showed the relationships of ticks, vertebrates and BBG, supports the hypothesis that the pathogens circulate through a nested and recursive network in almost closed clusters. In most of the clusters in which BBG circulates, there were one or more species of generalist ticks high values of centrality within the network that feed on a large range of phylogenetically diverse vertebrates. The specialist ticks in each cluster had low values of centrality and originated nested sub-networks that linked secondary vertebrates to the main nodes of the cluster in an expanding and branched web. Furthermore, vertebrates with higher positions in the range of centrality values tended to be more important in the circulation of BBG because they are the most prominent tick feeders. We postulate that two forces act in concert to produce the observed architecture of the BBG network: functional redundancy, i.e. the many vertebrates and ticks involved in pathogen circulation, and the co-evolutionary overlap of the environmental niches of ticks and vertebrates. Interactions between ticks and vertebrates were initially derived from their complementary ecological requirements, which increased the functional redundancy and allowed the pathogens to circulate. We hypothesise that sub-networks represent secondary niches between ticks and hosts that lead to subdivisions of the central core of the network due to expansion of the environmental dimensions of the interacting species or due to convergence of new species towards such niches. This functional redundancy may buffer the effects of the disturbance caused by the removal of vertebrates (i.e. the ticks are able to fill remaining biotic niches), thereby forming a highly resilient network. Although the analysis is incomplete, the results we obtained highlight the lack of sharing of BBG strains among the ticks. Further studies focusing on modelling the impact of removing vertebrate phylogenetic diversity on the adaptability of the system are necessary in order to identify a threshold at which the network would collapse.

## Additional files

Additional file 1: Complete set of data related to the associations of species of ticks, vertebrates and BBG as obtained by a literature search. (CSV 580 kb) Additional file 2: Phylogenetic tree of the genera of vertebrates in the epidemiological network of *B. burgdorferi* (BBG). The tree was generated using the available data from a mega-phylogeny and was computed and delivered by the Open Tree of Life (http://www.opentreeoflife.com/; accessed 19 Sept 2016). (PDF 50 kb) Additional file 3: The epidemiological network of *B. burgdorferi* (BBG) as visualised using the ForceAtlas2 algorithm at the level of the genera of vertebrates. The clusters are randomly coloured. Each cluster comprises a group of species that are more frequently reported to interact with each other, than with other species. The circles (nodes) represent organisms (species of pathogens and ticks or genera of vertebrates), and the lines with the same colour as a cluster represent interactions among the organisms of the cluster. The size of each node is proportional to its betweenness centrality, and the size of the label is proportional to the PageRank of the node. The width of each link is proportional to the weighted number of interactions between each pair of nodes. Although the network is directed, links lack arrows for improving visualization. (PDF 83 kb) Additional file 4: The epidemiological network of *B. burgdorferi* (BBG) as visualised using the ForceAtlas2 algorithm at the level of the species of vertebrates. The clusters are coloured randomly. Each cluster comprises a group of species that are more frequently reported to interact with each other, either as a parasite or as a transmitter, than with other species. The circles (nodes) represent organisms (species of pathogens and ticks or species of vertebrates), and the lines with the same colour interactions among organisms of the cluster. The size of the node is proportional to its betweenness centrality, and the size of the label is proportional to the PageRank of the node. The width of each link is proportional to the weighted number of interactions between each pair of nodes. Although the network is directed, links lack arrows for improving visualization. (PDF 139 kb) Additional file 5: Complete information for the ticks in the *B. burgdorferi* (BBG) epidemiological network. The table includes values of centrality (betweenness centrality, eigenvector centrality and PageRank), the clustering coefficient, the cluster in which the ticks circulate, the phylogenetic diversity (PD) of the hosts, the richness of the genera of hosts (GR), the circulation capacity (CC) for *B. burgdorferi*, the mean pairwise distances of hosts in the phylogenetic tree of the genera of hosts (MPD) and the significance of the MPD index (MPD-P). Negative MPD values and values of MPD-P near 0.01 indicate strong phylogenetic clustering of the hosts of the tick; positive MPD values and higher MPD-P values indicate weak phylogenetic clustering of the hosts of the tick. (CSV 15 kb) Additional file 6: Complete information for the *Borrelia* species in the *B. burgdorferi* (BBG) epidemiological network. The table includes values of centrality (betweenness centrality, eigenvector centrality and PageRank), the clustering coefficient, the cluster in which the ticks circulate, the phylogenetic diversity (PD) of the hosts, the richness of the genera of hosts (GR), the mean pairwise distances of hosts in the phylogenetic tree of the genera of hosts (MPD) and the significance of the MPD index (MPD-P). Negative MPD values and values of MPD-P near 0.01 indicate strong phylogenetic clustering of the hosts of the tick; positive MPD values and higher MPD-P values indicate weak phylogenetic clustering of the hosts of the tick. (CSV 7 kb) Additional file 7: Complete information for the genera of vertebrates (hosts and/or reservoirs) in the *B. burgdorferi* epidemiological network. The table includes values of centrality (betweenness centrality, eigenvector centrality and PageRank), the clustering coefficient, and the cluster in which the organisms circulate. (CSV 82 kb) Additional file 8: The records of ticks and species of *B. burgdorferi* (BBG) in the phylogenetic tree of the genera of vertebrates (see Additional file 2) for the significant clusters detected in the epidemiological network. Each blue circle represents a record of either a tick or a species of BBG on the host. A: cluster 1; B: cluster 2; C: cluster 5; D: cluster 6. (PDF 46 kb) Additional file 9: The phylogenetic tree for the concatenated multilocus sequence typing (MLST) sequences of *B. burgdorferi* (BBG) as recorded on the most represented ticks in the MLST dataset. The tip of each branch is labelled with the MLST classification. Each blue dot indicates that it was found in the reference tick. The size of the dot is proportional to the number of times that the specific MLST type was recorded on that tick. (PDF 272 kb) Additional file 10: The phylogenetic tree for the concatenated multilocus sequence typing (MLST) sequences of *B. burgdorferi* (BBG) as recorded on the most represented ticks in the MLST dataset. The tip of each branch is labelled with the MLST classification. Each blue dot indicates that it was found in the reference tick. The size of the dot is proportional to the number of times that the specific MLST type was recorded on that tick. (PDF 272 kb) Additional file 11: The phylogenetic tree for the concatenated multilocus sequence typing (MLST) sequences of *B. burgdorferi* (BBG) as recorded on the most represented ticks in the MLST dataset. The tip of each branch is labelled with the MLST classification. Each blue dot indicates that it was found in the reference tick. The size of the dot is proportional to the number of times that the specific MLST type was recorded on that tick. (PDF 272 kb) Additional file 12: The phylogenetic tree for the concatenated multilocus sequence typing (MLST) sequences of *B. burgdorferi* (BBG) as recorded on the most represented ticks in the MLST dataset. The tip of each branch is labelled with the MLST classification. Each blue dot indicates that it was found in the reference tick. The size of the dot is proportional to the number of times that the specific MLST type was recorded on that tick. (PDF 272 kb) Additional file 13: The phylogenetic tree for the concatenated multilocus sequence typing (MLST) sequences of *B. burgdorferi* (BBG) as recorded on the most represented ticks in the MLST dataset. The tip of each branch is labelled with the MLST classification. Each blue dot indicates that it was found in the reference tick. The size of the dot is proportional to the number of times that the specific MLST type was recorded on that tick. (PDF 272 kb) Additional file 14: The network of strains of *B. burgdorferi* (BBG), ticks and vertebrates as characterised by multilocus sequence typing (MLST) analysis using the ForceAtlas2 algorithm to determine the clusters and retaining only the strains recorded from questing ticks or hosts. This figure complements the data in Fig. 4. Each number represents a MLST strain that is linked to either the tick or the vertebrate where it was recorded. The width of the link is proportional to the weighted number of times that the link was recorded. The label size is proportional to the centrality of the node as measured using PageRank. The name of the species of BBG is also included in the link. The colour of the link represents the species of BBG. (PDF 4212 kb)

## Abbreviations

BBG: *Borrelia burgdorferi* (*s.l.*) group
BNC: Betweenness centrality
CC: Circulation capacity
MLST: Multilocus sequence typing
CCL: Chemokine ligand
PD: Faith’s phylogenetic diversity
PR: PageRank
WCC: Weighted clustering coefficient
WD: Weighted degree
