# Supplementary figures and images for: Nested coevolutionary networks shape the ecological relationships of ticks, hosts, and the Lyme disease bacteria of the Borrelia burgdorferi (s.l.) complex
Source: Parasit Vectors. 2016 Sep 23;9:517. doi: 10.1186/s13071-016-1803-z (PMC5035442; doi:10.1186/s13071-016-1803-z)

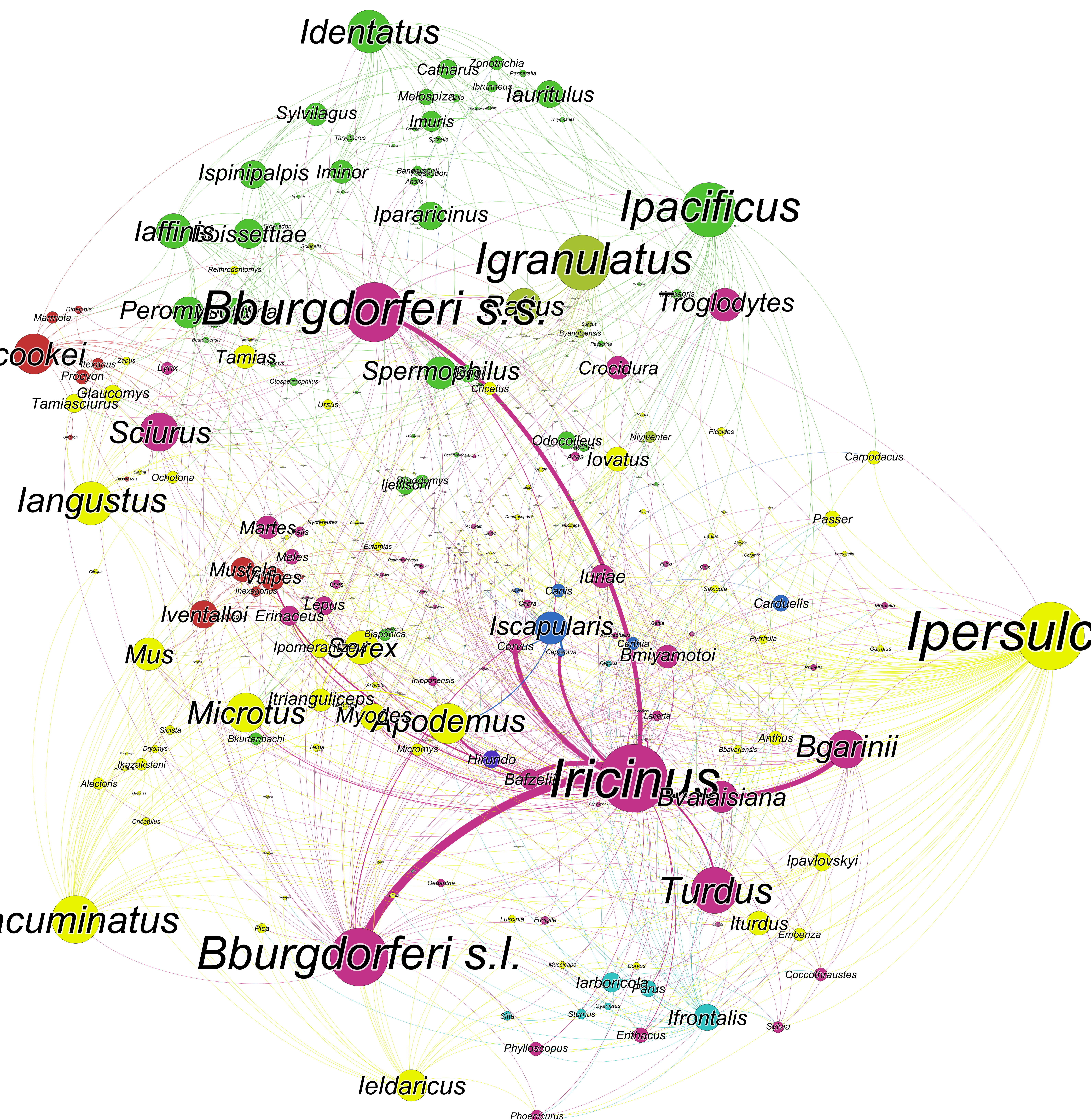

Supplement: Additional file 3: — The epidemiological network of B. burgdorferi (BBG) as visualised using the ForceAtlas2 algorithm at the level of the genera of vertebrates. The clusters are randomly coloured. Each cluster comprises a group of species that are more frequently reported to interact with each other, than with other species. The circles (nodes) represent organisms (species of pathogens and ticks or genera of vertebrates), and the lines with the same colour as a cluster represent interactions among the organisms of the cluster. The size of each node is proportional to its betweenness centrality, and the size of the label is proportional to the PageRank of the node. The width of each link is proportional to the weighted number of interactions between each pair of nodes. Although the network is directed, links lack arrows for improving visualization. (PDF 83 kb) [file 13071_2016_1803_MOESM3_ESM.pdf]

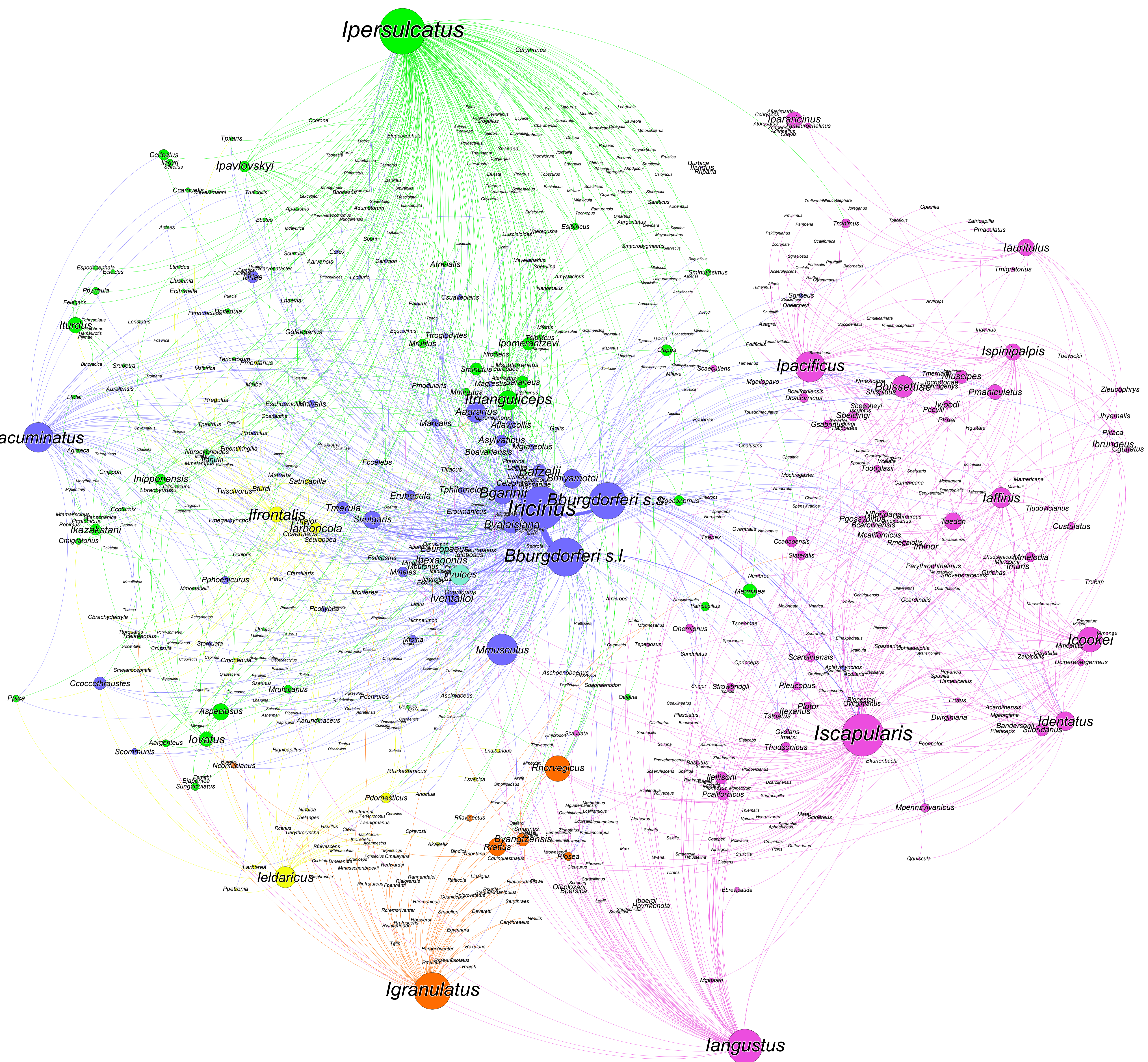

Supplement: Additional file 4: — The epidemiological network of B. burgdorferi (BBG) as visualised using the ForceAtlas2 algorithm at the level of the species of vertebrates. The clusters are coloured randomly. Each cluster comprises a group of species that are more frequently reported to interact with each other, either as a parasite or as a transmitter, than with other species. The circles (nodes) represent organisms (species of pathogens and ticks or species of vertebrates), and the lines with the same colour interactions among organisms of the cluster. The size of the node is proportional to its betweenness centrality, and the size of the label is proportional to the PageRank of the node. The width of each link is proportional to the weighted number of interactions between each pair of nodes. Although the network is directed, links lack arrows for improving visualization. (PDF 139 kb) [file 13071_2016_1803_MOESM4_ESM.pdf]

A

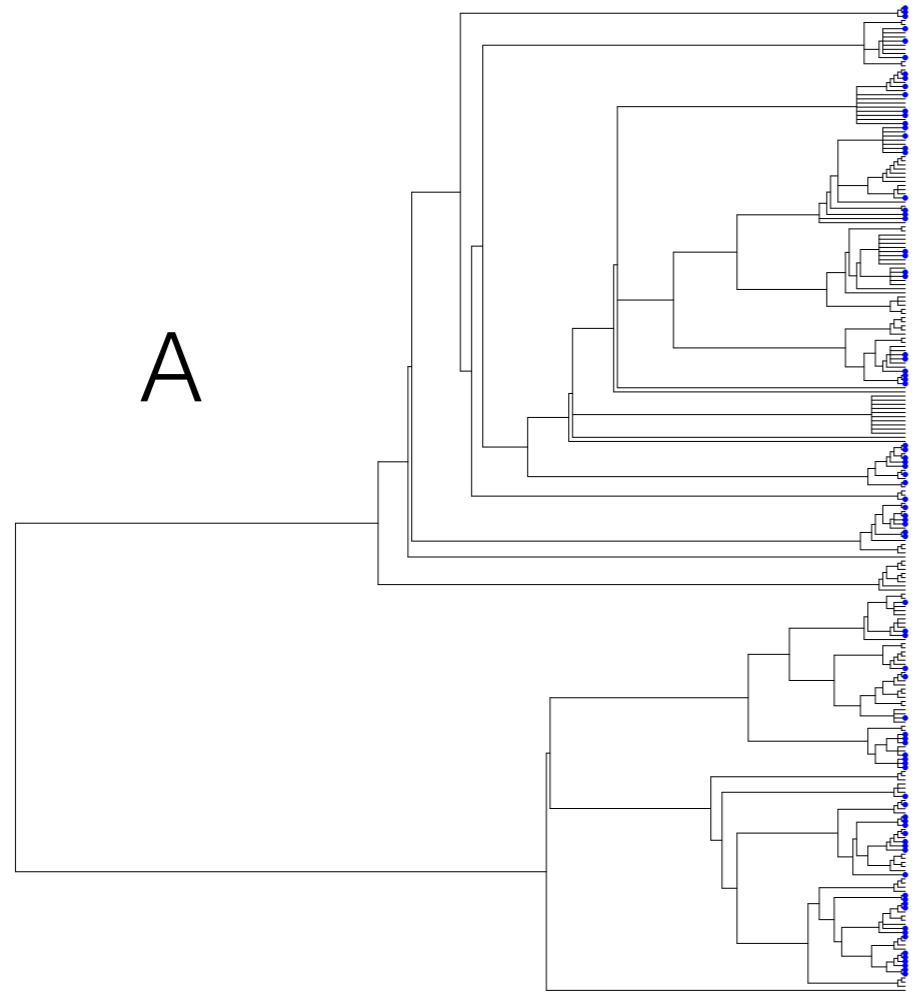

B

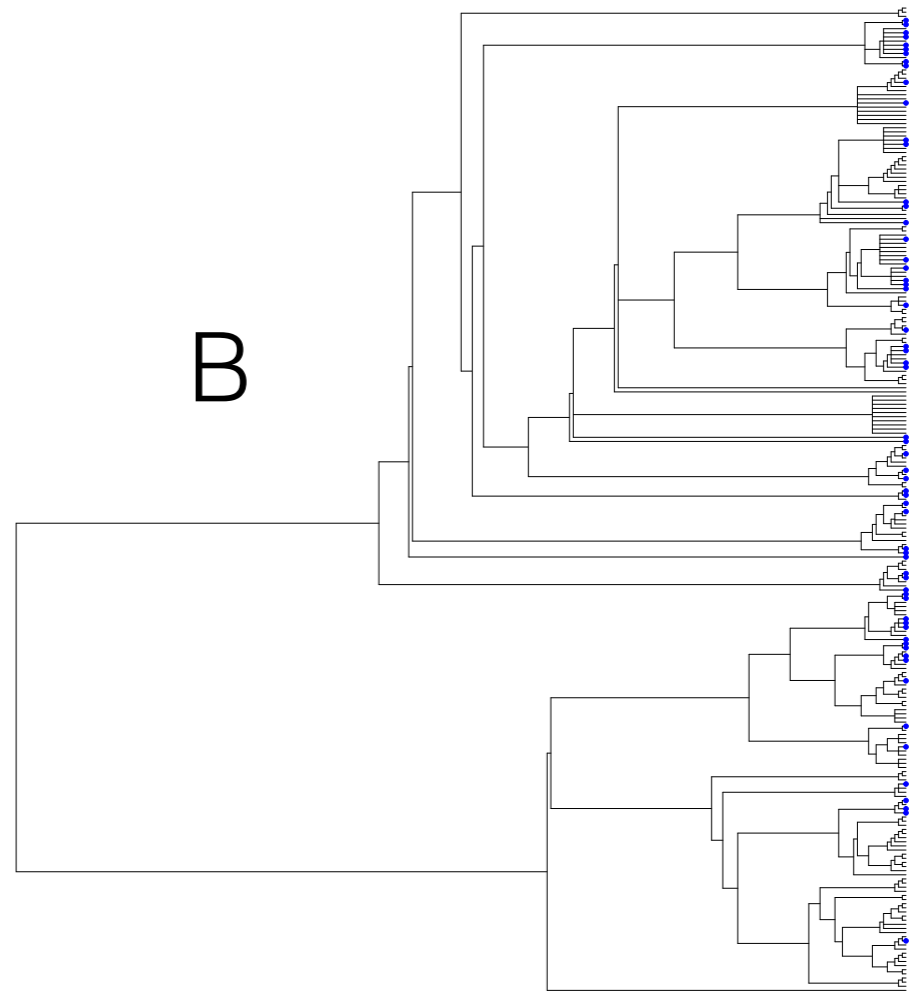

C

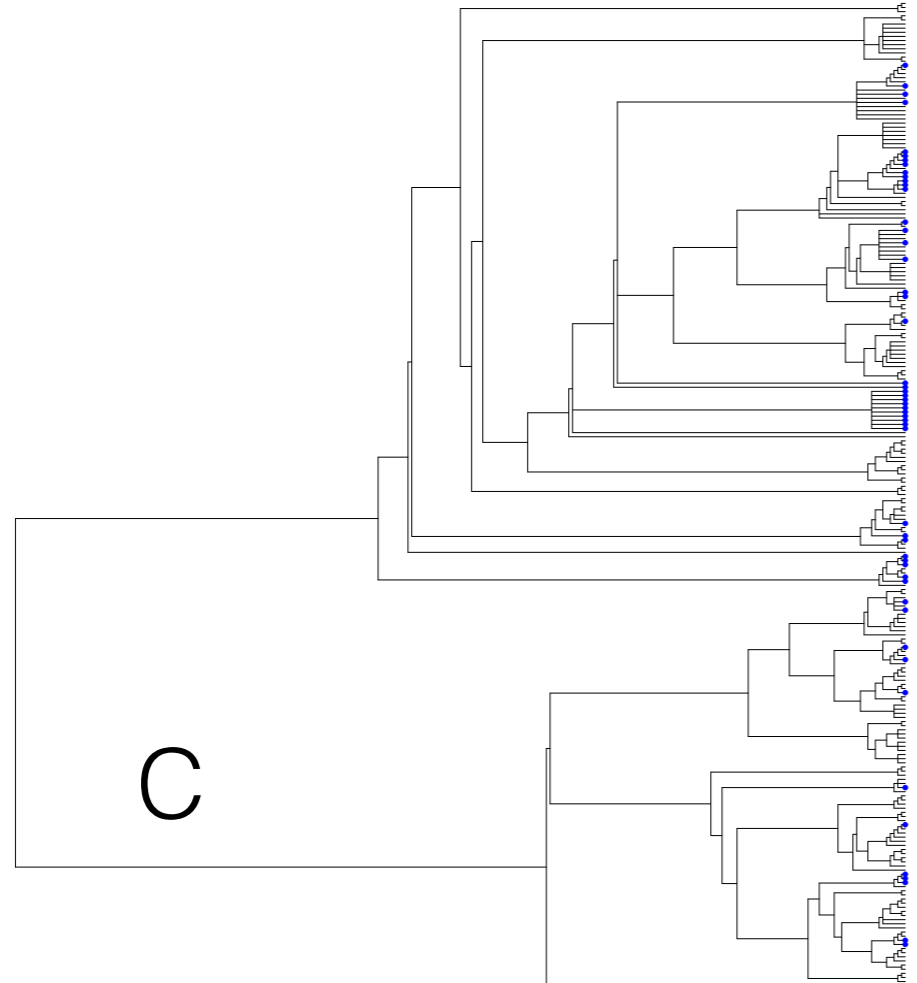

D

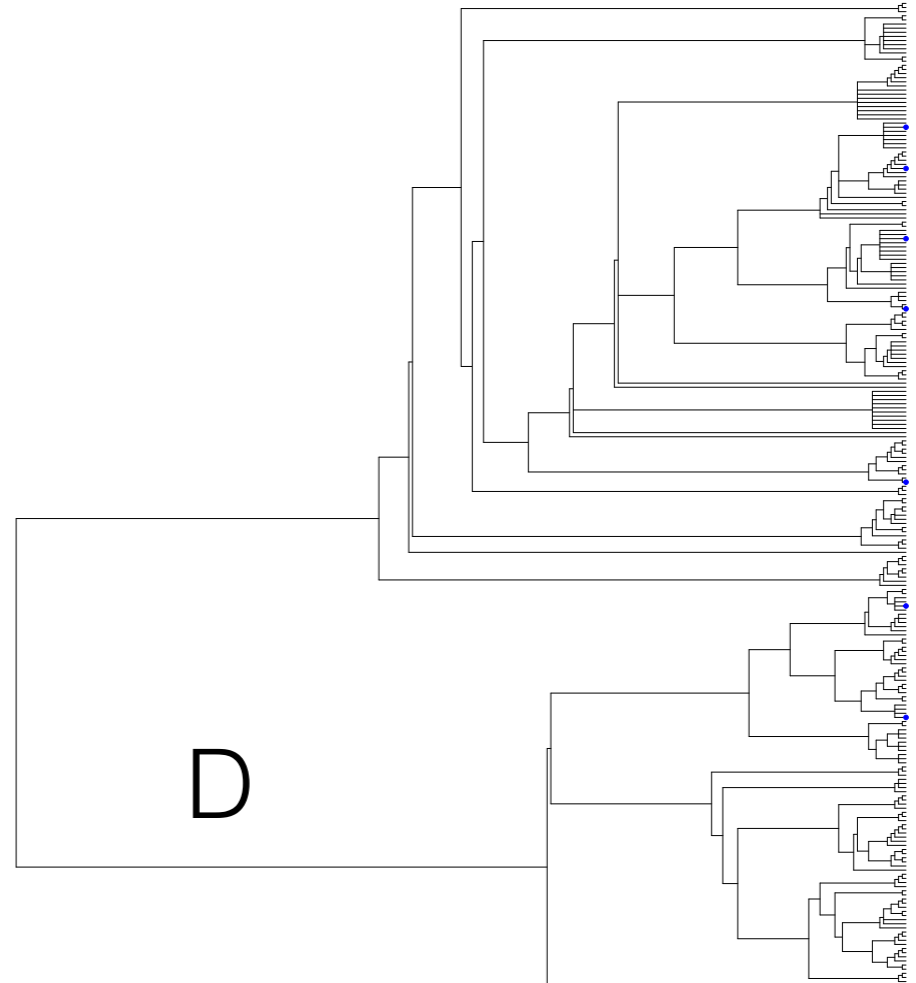

Supplement: Additional file 8: — The records of ticks and species of B. burgdorferi (BBG) in the phylogenetic tree of the genera of vertebrates (see Additional file 2) for the significant clusters detected in the epidemiological network. Each blue circle represents a record of either a tick or a species of BBG on the host. A: cluster 1; B: cluster 2; C: cluster 5; D: cluster 6. (PDF 46 kb) [file 13071_2016_1803_MOESM8_ESM.pdf]

*Ixodes pacificus*

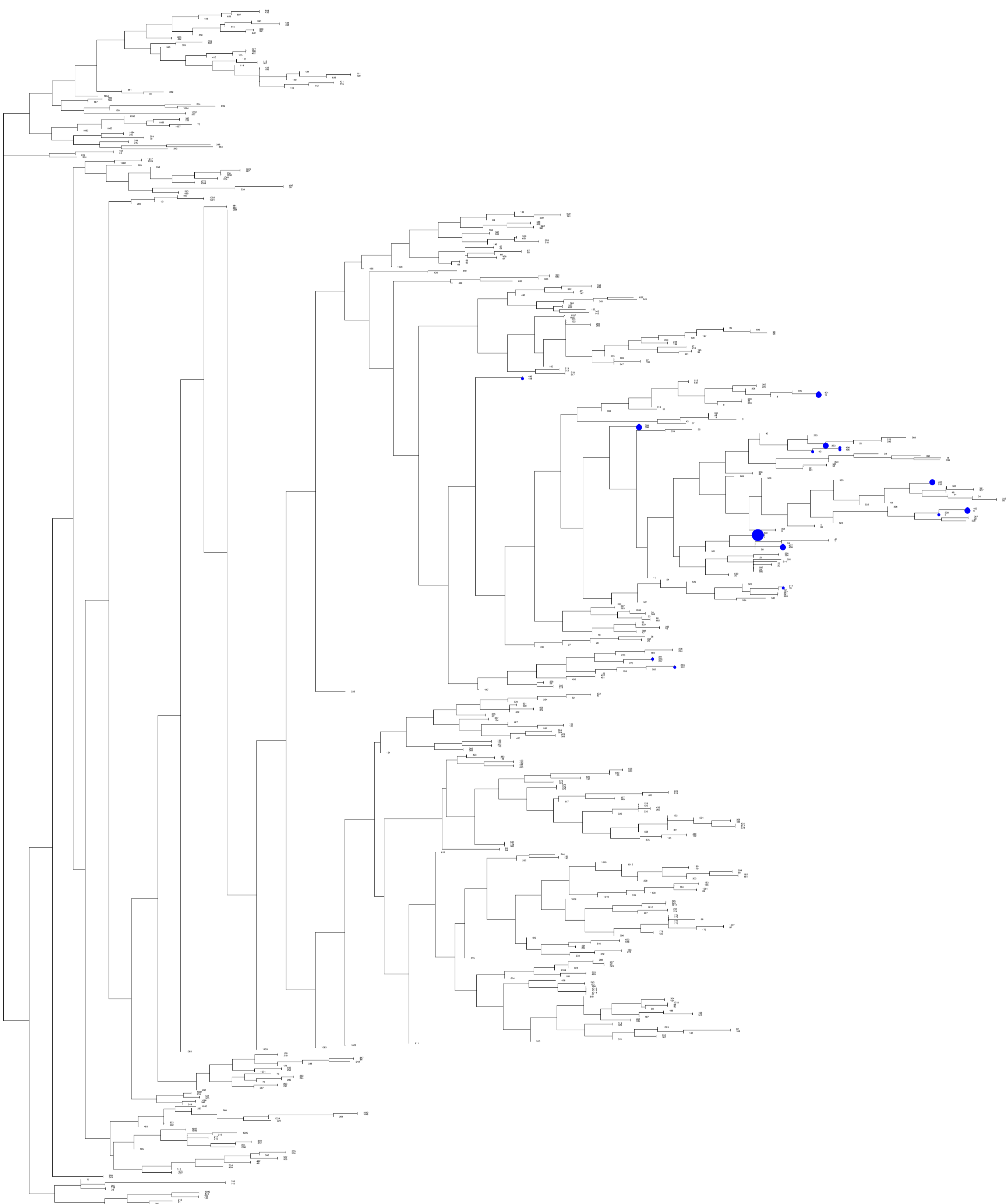

Supplement: Additional file 9: — The phylogenetic tree for the concatenated multilocus sequence typing (MLST) sequences of B. burgdorferi (BBG) as recorded on the most represented ticks in the MLST dataset. The tip of each branch is labelled with the MLST classification. Each blue dot indicates that it was found in the reference tick. The size of the dot is proportional to the number of times that the specific MLST type was recorded on that tick. (PDF 272 kb) [file 13071_2016_1803_MOESM9_ESM.pdf]

*Ixodes pavlovskyi*

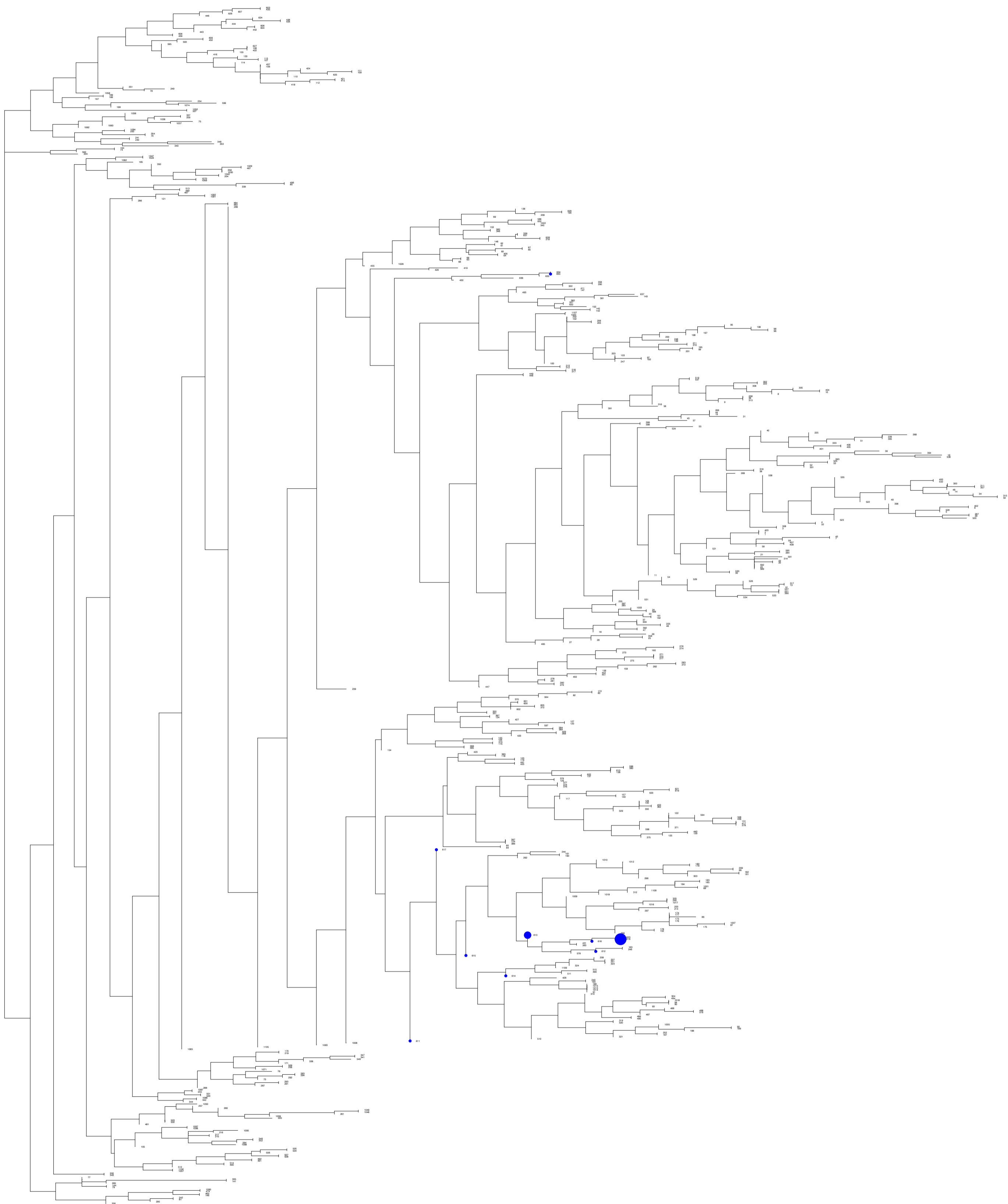

Supplement: Additional file 10: — The phylogenetic tree for the concatenated multilocus sequence typing (MLST) sequences of B. burgdorferi (BBG) as recorded on the most represented ticks in the MLST dataset. The tip of each branch is labelled with the MLST classification. Each blue dot indicates that it was found in the reference tick. The size of the dot is proportional to the number of times that the specific MLST type was recorded on that tick. (PDF 272 kb) [file 13071_2016_1803_MOESM10_ESM.pdf]

*Ixodes persulcatus*

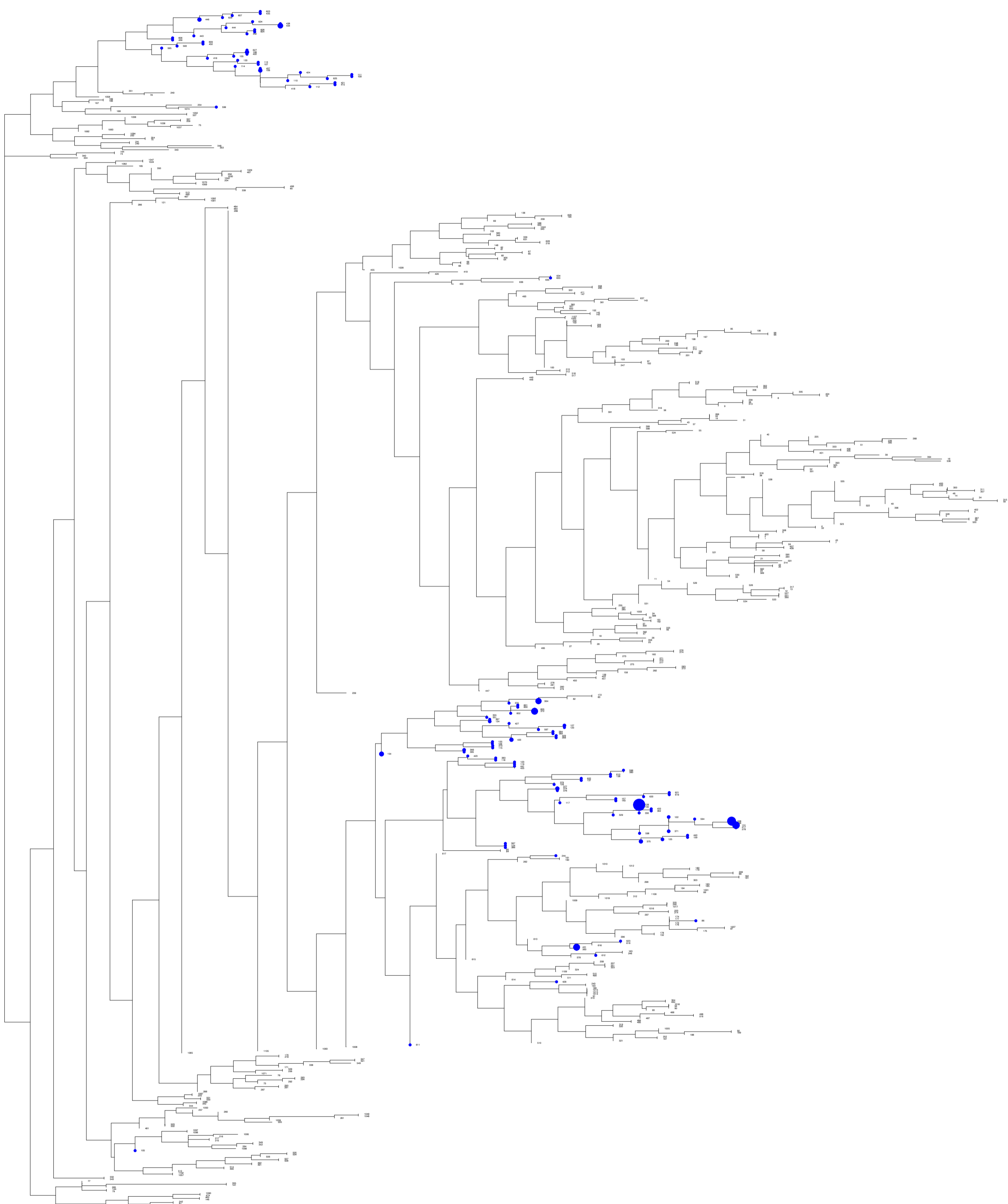

Supplement: Additional file 11: — The phylogenetic tree for the concatenated multilocus sequence typing (MLST) sequences of B. burgdorferi (BBG) as recorded on the most represented ticks in the MLST dataset. The tip of each branch is labelled with the MLST classification. Each blue dot indicates that it was found in the reference tick. The size of the dot is proportional to the number of times that the specific MLST type was recorded on that tick. (PDF 272 kb) [file 13071_2016_1803_MOESM11_ESM.pdf]

*Ixodes ricinus*

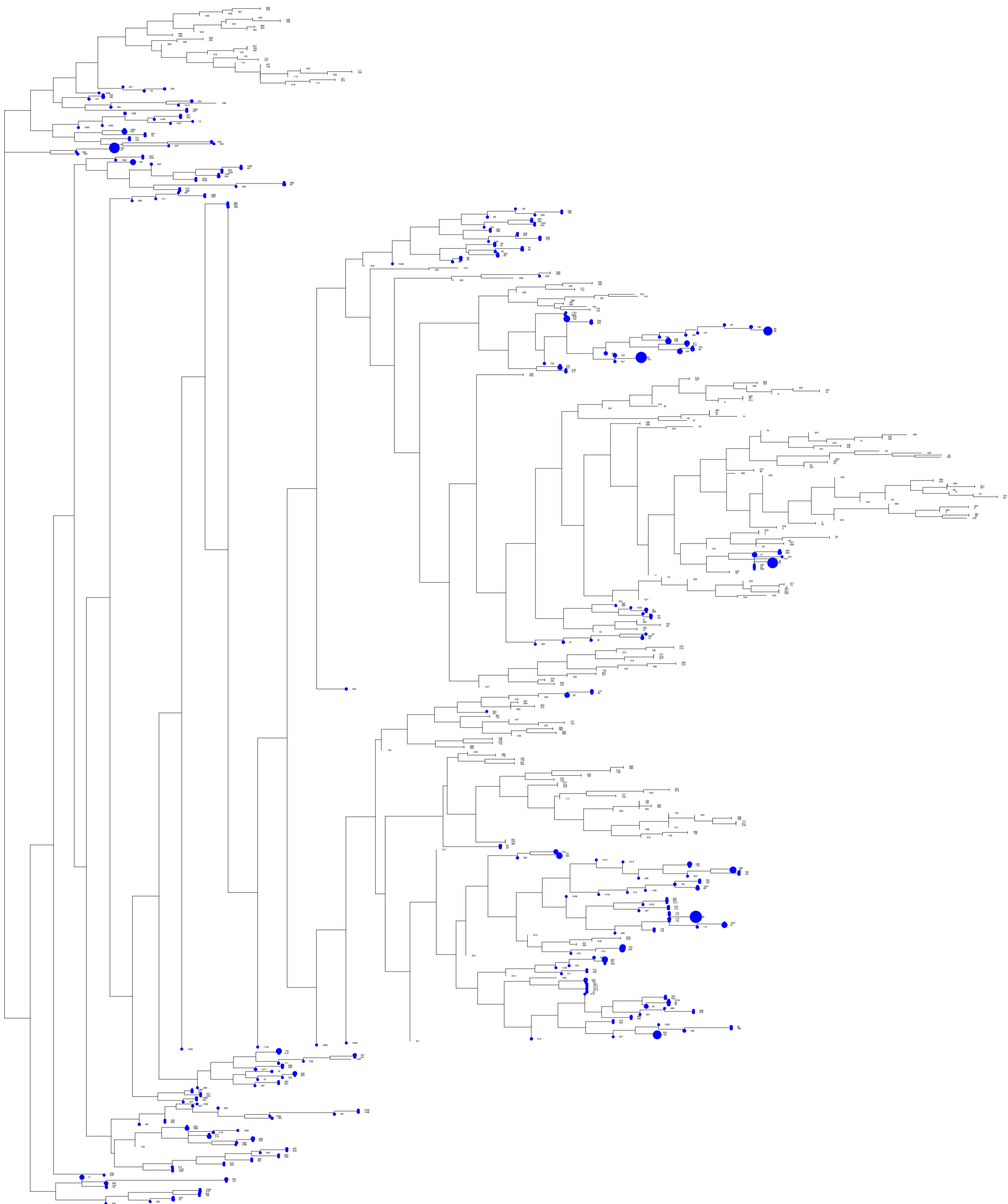

Supplement: Additional file 12: — The phylogenetic tree for the concatenated multilocus sequence typing (MLST) sequences of B. burgdorferi (BBG) as recorded on the most represented ticks in the MLST dataset. The tip of each branch is labelled with the MLST classification. Each blue dot indicates that it was found in the reference tick. The size of the dot is proportional to the number of times that the specific MLST type was recorded on that tick. (PDF 272 kb) [file 13071_2016_1803_MOESM12_ESM.pdf]

*Ixodes scapularis*

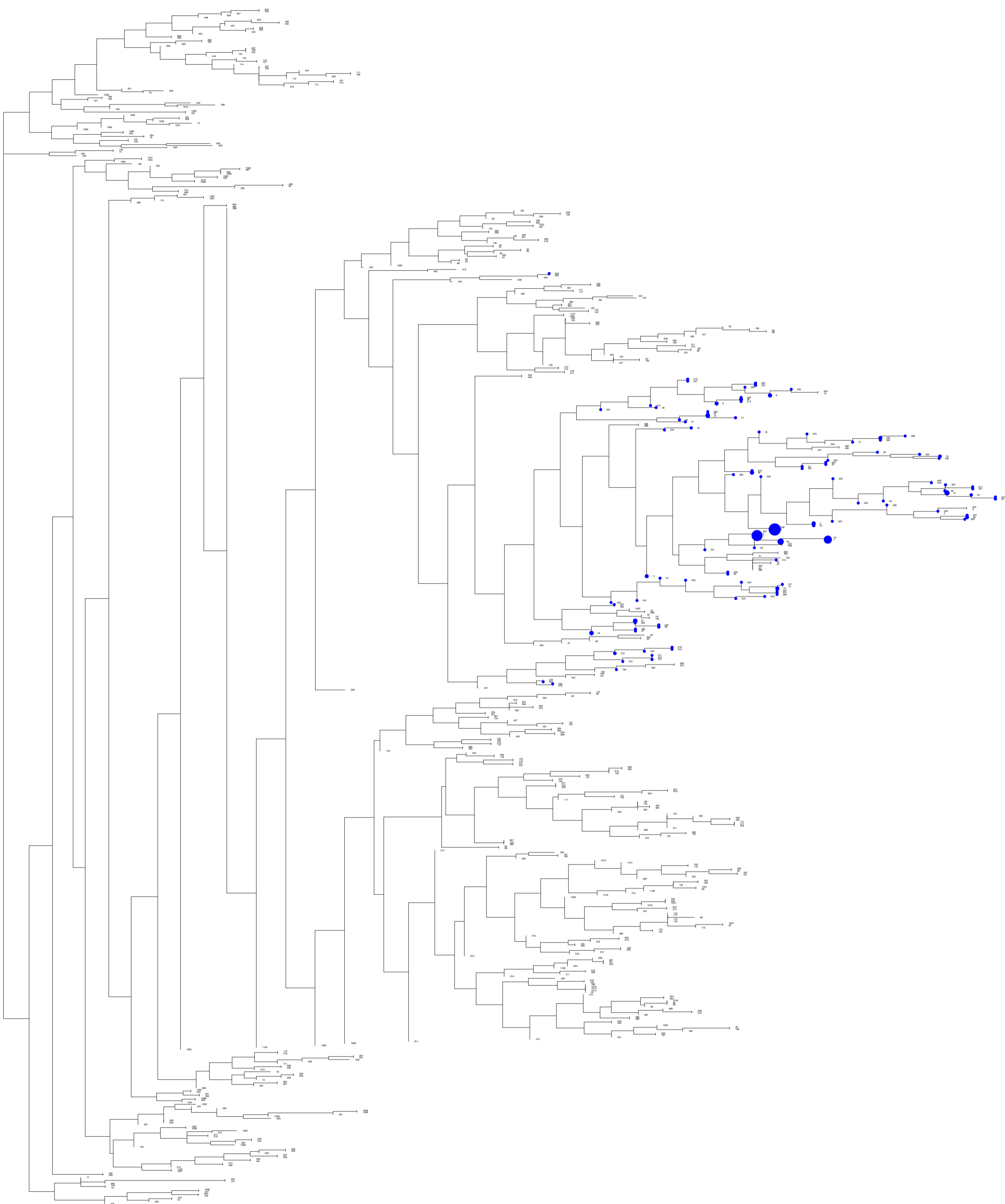

Supplement: Additional file 13: — The phylogenetic tree for the concatenated multilocus sequence typing (MLST) sequences of B. burgdorferi (BBG) as recorded on the most represented ticks in the MLST dataset. The tip of each branch is labelled with the MLST classification. Each blue dot indicates that it was found in the reference tick. The size of the dot is proportional to the number of times that the specific MLST type was recorded on that tick. (PDF 272 kb) [file 13071_2016_1803_MOESM13_ESM.pdf]

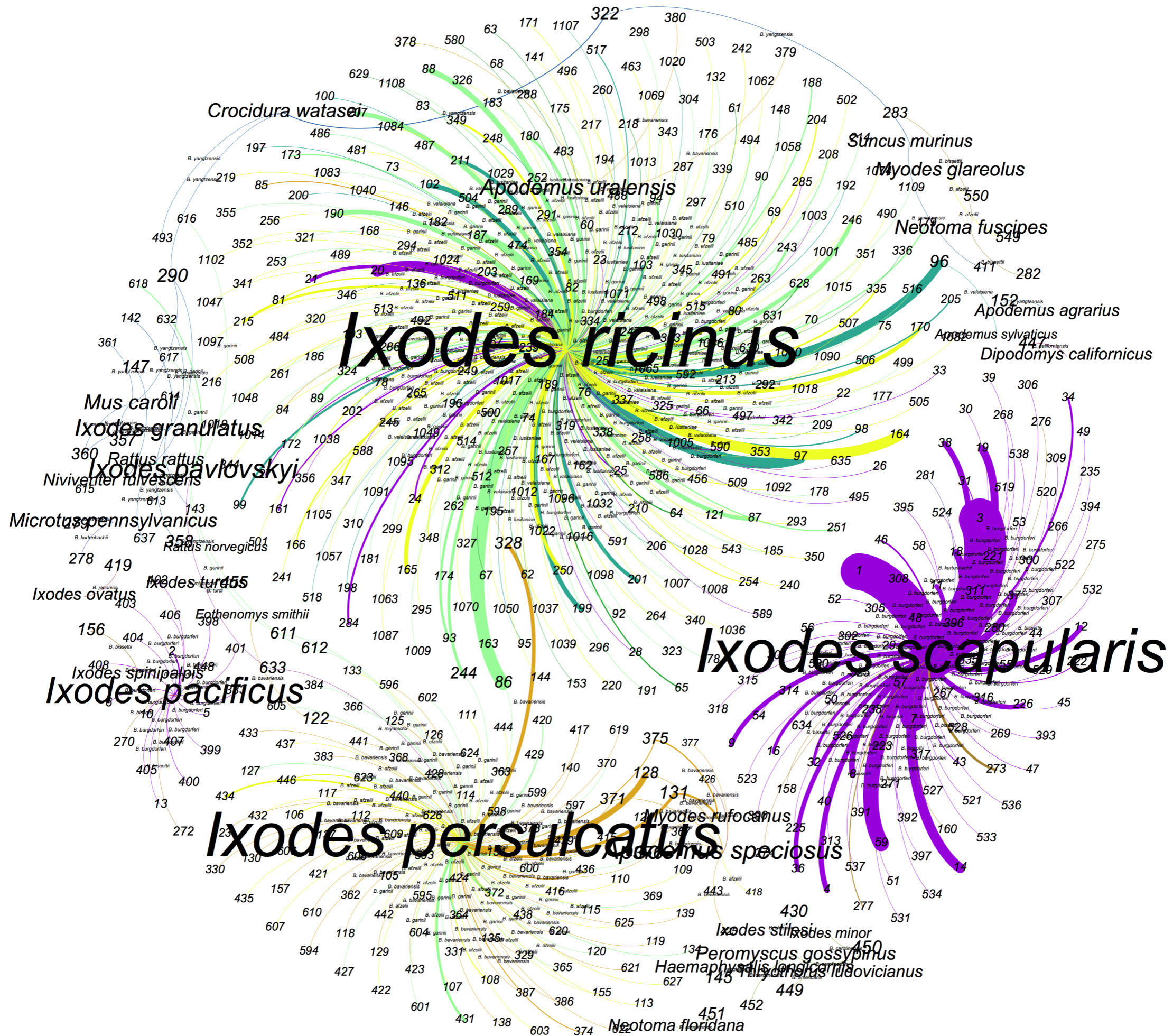

Supplement: Additional file 14: — The network of strains of B. burgdorferi (BBG), ticks and vertebrates as characterised by multilocus sequence typing (MLST) analysis using the ForceAtlas2 algorithm to determine the clusters and retaining only the strains recorded from questing ticks or hosts. This figure complements the data in Fig. 4. Each number represents a MLST strain that is linked to either the tick or the vertebrate where it was recorded. The width of the link is proportional to the weighted number of times that the link was recorded. The label size is proportional to the centrality of the node as measured using PageRank. The name of the species of BBG is also included in the link. The colour of the link represents the species of BBG. (PDF 4212 kb) [file 13071_2016_1803_MOESM14_ESM.pdf]
