# Supplementary material for: Nested coevolutionary networks shape the ecological relationships of ticks, hosts, and the Lyme disease bacteria of the Borrelia burgdorferi (s.l.) complex
Source: Parasit Vectors. 2016 Sep 23;9:517. doi: 10.1186/s13071-016-1803-z (PMC5035442; doi:10.1186/s13071-016-1803-z)
Supplement: Additional file 2: — Phylogenetic tree of the genera of vertebrates in the epidemiological network of B. burgdorferi (BBG). The tree was generated using the available data from a mega-phylogeny and was computed and delivered by the Open Tree of Life (http://www.opentreeoflife.com/; accessed 19 Sept 2016). (PDF 50 kb) [file 13071_2016_1803_MOESM2_ESM.pdf]

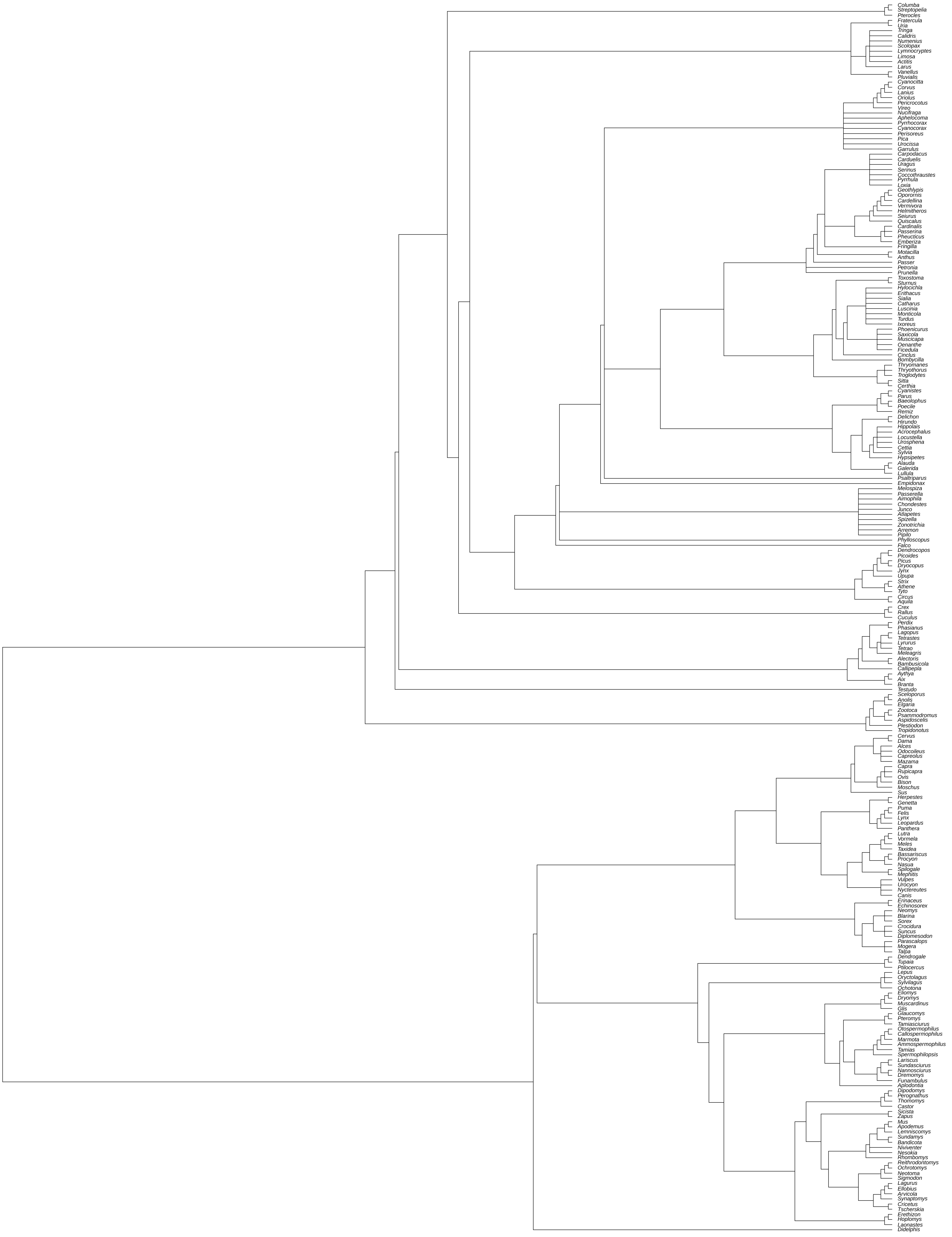
Columba
Streptopelia
Pterocles
Fratercula
Uria
Tringa
Calidris
Numenius
Scolopax
Lymnocryptes
Limosa
Actitis
Larus
Vanellus
Pluvialis
Cyanocitta
Corvus
Lanius
Oriolus
Pericrocotus
Vireo
Nucifraga
Aphelocoma
Pyrrhocorax
Cyanocorax
Perisoreus
Pica
Urocissa
Garrulus
Carpodacus
Carduelis
Uragus
Serinus
Coccothraustes
Pyrrhula
Loxia
Geothlypis
Oporornis
Cardellina
Vermivora
Helmitheros
Seiurus
Quiscalus
Cardinalis
Passerina
Pheucticus
Emberiza
Fringilla
Motacilla
Anthus
Passer
Petronia
Prunella
Toxostoma
Sturnus
Hylocichla
Erithacus
Sialia
Catharus
Luscinia
Monticola
Turdus
Ixoreus
Phoenicurus
Saxicola
Muscicapa
Oenanthe
Ficedula
Cinclus
Bombycilla
Thryomanes
Thryothorus
Troglodytes
Sitta
Certhia
Cyanistes
Parus
Baeolophus
Poecile
Remiz
Delichon
Hirundo
Hippolais
Acrocephalus
Locustella
Urosphena
Cettia
Sylvia
Hypsipetes
Alauda
Galerida
Lullula
Psaltriparus
Empidonax
Melospiza
Passerella
Aimophila
Chondestes
Junco
Atlapetes
Spizella
Zonotrichia
Arremon
Pipilo
Phylloscopus
Falco
Dendrocopos
Picoides
Picus
Dryocopus
Jynx
Upupa
Strix
Athene
Tyto
Circus
Aquila
Crex
Rallus
Cuculus
Perdix
Phasianus
Lagopus
Tetrastes
Lyrurus
Tetrao
Meleagris
Alectoris
Bambusicola
Callipepla
Aythya
Aix
Branta
Testudo
Sceloporus
Anolis
Elgaria
Zootoca
Psammodromus
Aspidoscelis
Plestiodon
Tropidonotus
Cervus
Dama
Alces
Odocoileus
Capreolus
Mazama
Capra
Rupicapra
Ovis
Bison
Moschus
Sus
Herpestes
Genetta
Puma
Felis
Lynx
Leopardus
Panthera
Lutra
Vormela
Meles
Taxidea
Bassariscus
Procyon
Nasua
Spilogale
Mephitis
Vulpes
Urocyon
Nyctereutes
Canis
Erinaceus
Echinosorex
Neomys
Blarina
Sorex
Crocidura
Suncus
Diplomesodon
Parascalops
Mogera
Talpa
Dendrogale
Tupaia
Ptilocercus
Lepus
Oryctolagus
Sylvilagus
Ochotona
Eliomys
Dryomys
Muscardinus
Glis
Glaucomys
Pteromys
Tamiasciurus
Otospermophilus
Callospermophilus
Marmota
Ammospermophilus
Tamias
Spermophilopsis
Lariscus
Sundasciurus
Nannosciurus
Dremomys
Funambulus
Aplodontia
Dipodomys
Perognathus
Thomomys
Castor
Sicista
Zapus
Mus
Apodemus
Lemniscomys
Sundamys
Bandicota
Niviventer
Nesokia
Rhombomys
Reithrodontomys
Ochrotomys
Neotoma
Sigmodon
Lagurus
Ellobius
Arvicola
Synaptomys
Cricetus
Tscherskia
Erethizon
Hoplomys
Laonastes
Didelphis
